# Physiological and pathophysiological mechanisms of the molecular and cellular biology of angiogenesis and inflammation in moyamoya angiopathy and related vascular diseases

**DOI:** 10.3389/fneur.2023.661611

**Published:** 2023-05-16

**Authors:** Kirsten B. Dorschel, John E. Wanebo

**Affiliations:** ^1^Medical Faculty, Heidelberg University Medical School, Ruprecht-Karls-Universität Heidelberg, Heidelberg, Germany; ^2^Department of Neurosurgery, Barrow Neurological Institute, St. Joseph's Hospital and Medical Center, Phoenix, AZ, United States; ^3^Department of Neuroscience, HonorHealth Research Institute, Scottsdale, AZ, United States

**Keywords:** moyamoya angiopathy (MMA), molecular mechanism, pathophysiology, angiogenesis, inflammation, genetics, stroke, biomarker

## Abstract

**Rationale:**

The etiology and pathophysiological mechanisms of moyamoya angiopathy (MMA) remain largely unknown. MMA is a progressive, occlusive cerebrovascular disorder characterized by recurrent ischemic and hemorrhagic strokes; with compensatory formation of an abnormal network of perforating blood vessels that creates a collateral circulation; and by aberrant angiogenesis at the base of the brain. Imbalance of angiogenic and vasculogenic mechanisms has been proposed as a potential cause of MMA. Moyamoya vessels suggest that aberrant angiogenic, arteriogenic, and vasculogenic processes may be involved in the pathophysiology of MMA. Circulating endothelial progenitor cells have been hypothesized to contribute to vascular remodeling in MMA. MMA is associated with increased expression of angiogenic factors and proinflammatory molecules. Systemic inflammation may be related to MMA pathogenesis.

**Objective:**

This literature review describes the molecular mechanisms associated with cerebrovascular dysfunction, aberrant angiogenesis, and inflammation in MMA and related cerebrovascular diseases along with treatment strategies and future research perspectives.

**Methods and results:**

References were identified through a systematic computerized search of the medical literature from January 1, 1983, through July 29, 2022, using the PubMed, EMBASE, BIOSIS Previews, CNKI, ISI web of science, and Medline databases and various combinations of the keywords “moyamoya,” “angiogenesis,” “anastomotic network,” “molecular mechanism,” “physiology,” “pathophysiology,” “pathogenesis,” “biomarker,” “genetics,” “signaling pathway,” “blood-brain barrier,” “endothelial progenitor cells,” “endothelial function,” “inflammation,” “intracranial hemorrhage,” and “stroke.” Relevant articles and supplemental basic science articles almost exclusively published in English were included. Review of the reference lists of relevant publications for additional sources resulted in 350 publications which met the study inclusion criteria. Detection of growth factors, chemokines, and cytokines in MMA patients suggests the hypothesis of aberrant angiogenesis being involved in MMA pathogenesis. It remains to be ascertained whether these findings are consequences of MMA or are etiological factors of MMA.

**Conclusions:**

MMA is a heterogeneous disorder, comprising various genotypes and phenotypes, with a complex pathophysiology. Additional research may advance our understanding of the pathophysiology involved in aberrant angiogenesis, arterial stenosis, and the formation of moyamoya collaterals and anastomotic networks. Future research will benefit from researching molecular pathophysiologic mechanisms and the correlation of clinical and basic research results.

## Introduction

**Figure 1:**
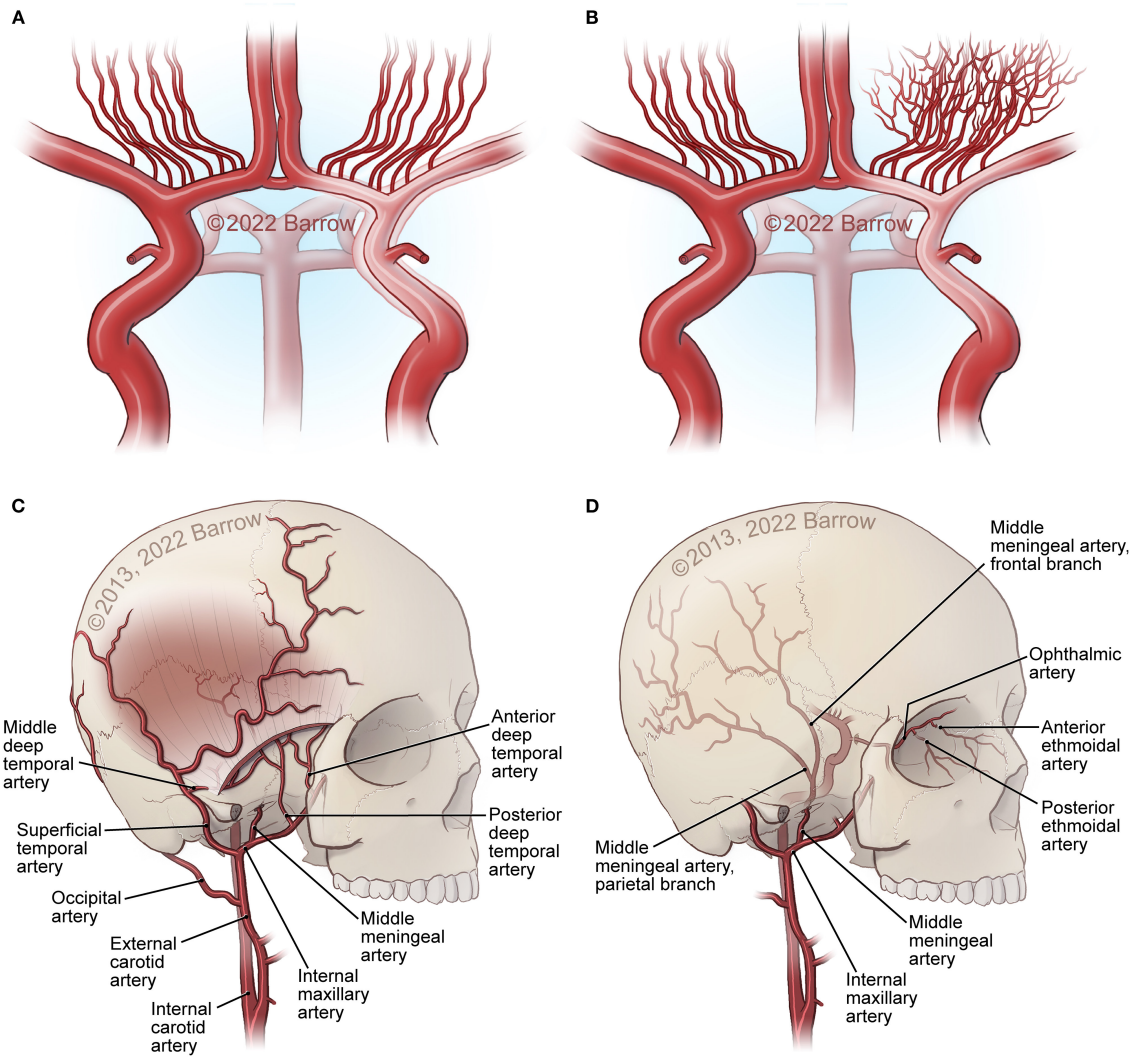
Major structural changes in MMA. **(A)** Carotid fork occlusion. **(B)** Development of moyamoya collaterals at the base of the brain. **(C, D)** External carotid artery (ECA) collaterals from the superficial temporal artery (STA), internal maxillary artery, ophthalmic artery ethmoidal artery and collaterals through foramina and sutures in the bone at the skull base and the surface of the skull, as well as middle meningeal artery connections from the dura into the middle cerebral artery (MCA) and anterior cerebral artery (ACA) surface branches. See Supplementary material. *Used with permission from Barrow Neurological Institute, Phoenix, Arizona*.

**Figure 2:**
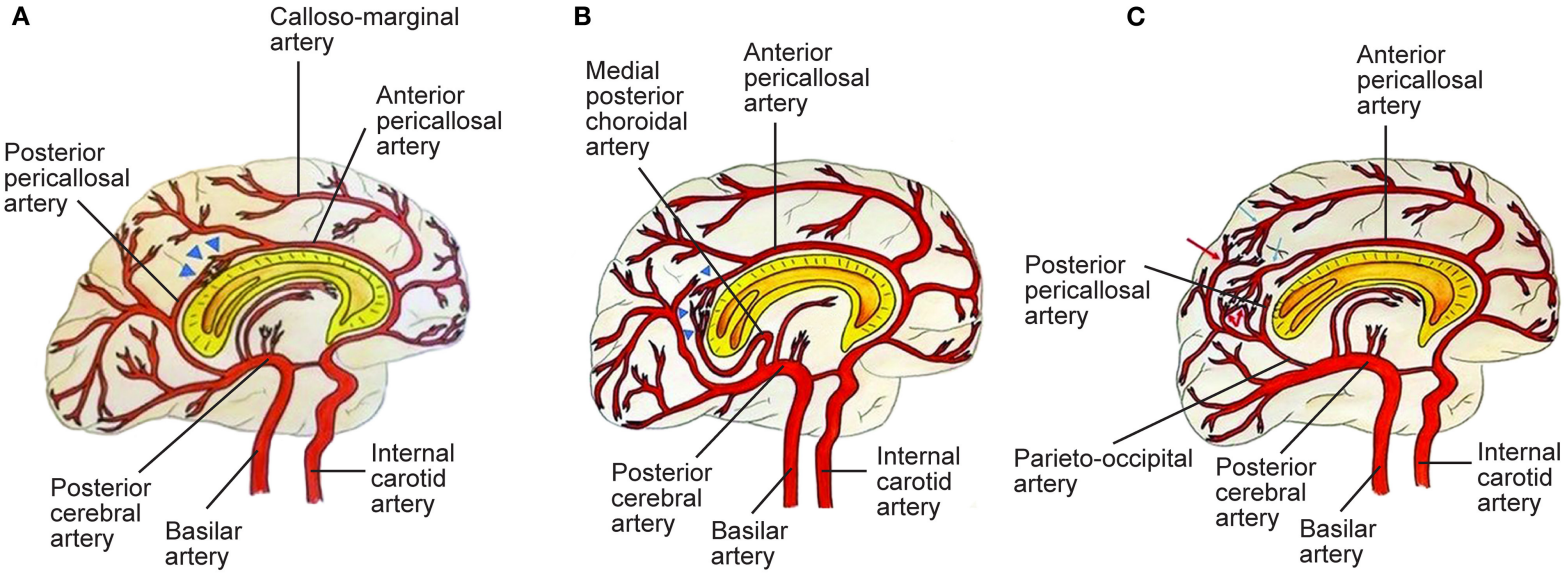
Three types of posterior cerebral artery (PCA)-anterior cerebral artery (ACA) collaterals. **(A)** Type I collaterals constitute the anastomosis between the anterior pericallosal artery (APA) and the posterior pericallosal artery (PPA) which is indicated by blue triangles. Pio-pial connections contribute to this anastomosis. **(B)** Type II collaterals between the APA and the medial posterior choroidal artery (MPChoA) are indicated by blue triangles. The MPChoA first turns anteriorly and then backwards around the splenium of the corpus callosum toward the APA. **(C)** Type III collaterals are leptomeningeal or pio-pial connections between cortical branches from the PCA, indicated by red arrows, and cortical branches from the ACA, indicated by light blue arrows. PPA-APA connections are present (7). See Supplementary material. *Source: Reprinted/adapted from Bonasia et al*. *(**7**)* *with permission from the AJNR, American Journal of Neuroradiology, American Society of Neuroradiology, and American Roentgen Ray Society*.

**Figure 3:**
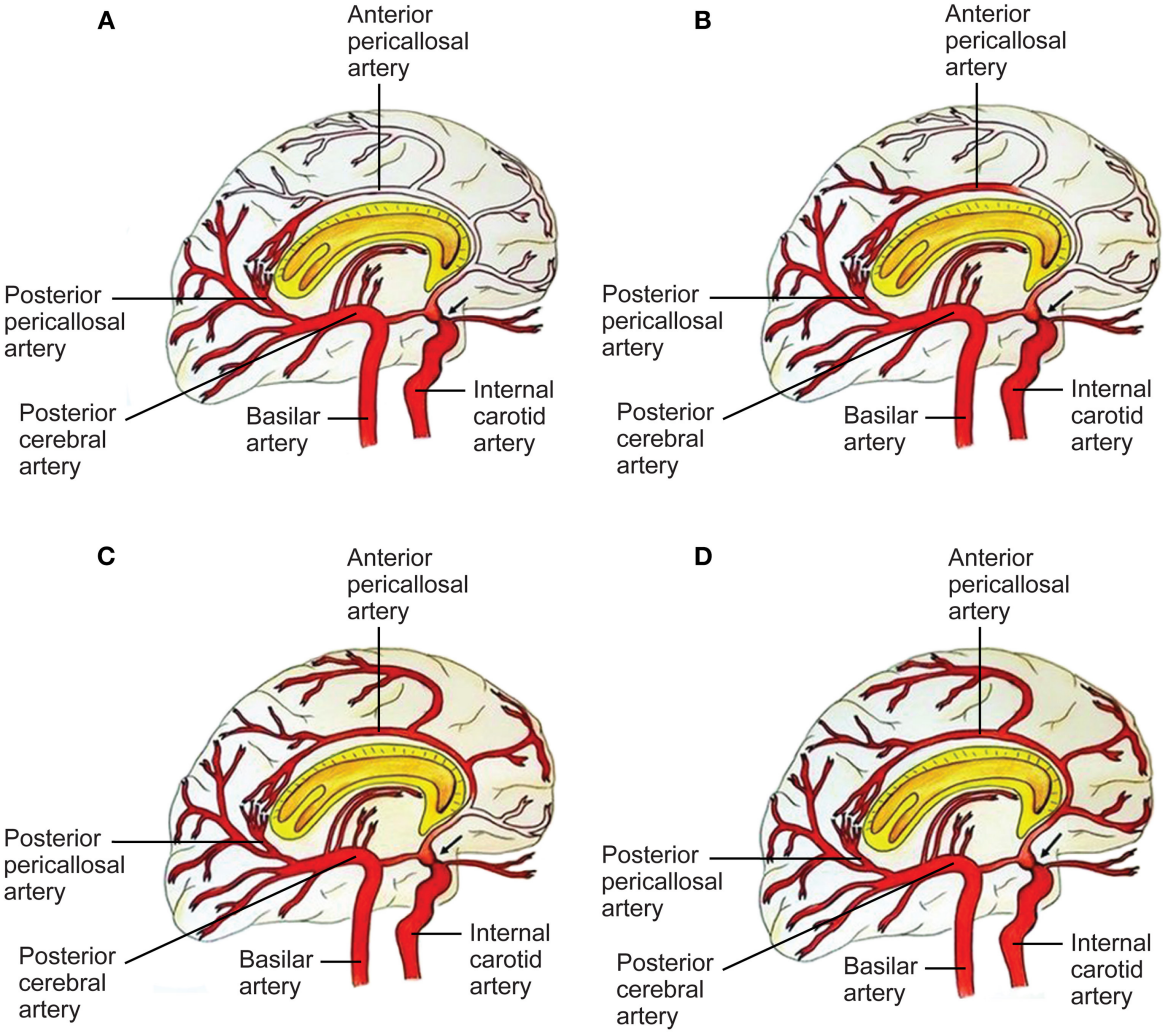
Capacity to compensate the ACA territory through posterior cerebral artery (PCA)-anterior cerebral artery (ACA) anastomoses in proximal ICA stenosis (black arrow). A four-grade classification. **(A)** In grade I, collaterals refill the first part of the ACA, without any cortical branches. Pio-pial connections and the posterior pericallosal artery contribute to refilling. **(B)** In grade II, the retrograde flow reaches a larger part of the ACA, including a cortical branch of the ACA. A contribution from the medial posterior choroidal artery and the pio-pial connection may be involved. **(C)** Grade III consists of retrograde refilling of three or two ACA branches, which may be strengthened by e.g., a medial posterior choroidal artery–anterior pericallosal artery anastomosis. **(D)** In grade IV, retrograde refilling reaches nearly the entire ACA territory. Major distinct connections may compensate the hypoperfusion of the ACA territory (7). See Supplementary material. *Source: Reprinted/adapted from Bonasia et al*. *(**7**)* *with permission from the AJNR, American Journal of Neuroradiology, American Society of Neuroradiology, and American Roentgen Ray Society*.

Moyamoya angiopathy (MMA) is an angiopathy unique to the cerebrovasculature that is characterized by chronically progredient stenosis of the bilateral intracranial internal carotid artery (ICA) and its proximal bifurcations and development of a network of aberrant collateral arteries to compensate for the stenosed vessels. MMA pathophysiology may include a consecutive secondary response of compensatory collateral circulation development by means of vasculogenesis and alteration of cerebral hemodynamics as a result of a primary narrowing of distinct intracranial vessels (1–6) (Figures 1–3) (see the Supplementary Table 1 for definitions of gene symbols, proteins, and additional terminology).

Comprehension of cellular signaling cascades linked to MMA may be essential for identifying diagnostic and therapeutic targets (5). Distinct monogenic moyamoya syndromes show radiological characteristics of MMA and may be related to various signaling pathways and genes associated with MMA pathogenesis (8). Through identification of genes involved in MMA pathogenesis and several monogenic moyamoya syndromes (MMS), researchers have associated various signaling pathways with MMA pathophysiology, including molecular signaling pathways [Rat sarcoma (Ras)–rat fibrosarcoma (Raf)–mitogen-activated protein kinase (MEK)–extracellular signal-related kinase (ERK) signaling pathway, nitric oxide (NO)–soluble guanylyl cyclase (sGC)–cyclic guanosine monophosphate (cGMP) signaling pathway], signaling pathways involved in inflammation [Phosphatidylinositol 3-kinase (PI3K)/and Akt1 (Akt)/mammalian target of rapamycin (mTOR) signaling pathway, hypoxia-inducible factor (HIF)-1/nuclear factor kappa-light-chain-enhancer of activated B cells (NF-κB) signaling pathway, Caveolin-1/ERK signaling pathway, the wingless and Int-1 (Wnt)/(β-Catenin)/lymphoid enhancing factor (Lef)-1 signaling pathway, Calcineurein/nuclear factor of activated T-cells (NFAT) signaling pathway, mitogen-activated protein kinase (MAPK) signaling pathway, tumor necrosis factor alpha (TNFα)/protein tyrosine phosphatase 1B (PTP1B) and peroxisome proliferator activated receptor gamma (PPARγ) signaling pathway, toll-like receptor (TLR) signaling pathway], and signaling pathways involved in genomic stability [Ring finger protein 213 (RNF213) signaling pathway]. Genes encoding additional members of these pathways may themselves be involved in MMA pathogenesis (3, 9–11). Inflammatory proteins have been shown to be associated with MMA pathophysiology. However, inflammatory proteins have not been historically approved as causative agents of MMA (5, 12). Research into physiologic characteristics of angiogenesis, arteriogenesis, vasculogenesis, and associated signaling pathways may lead to a deeper understanding of moyamoya's complex pathophysiology (13–42).

**Figure 4:**
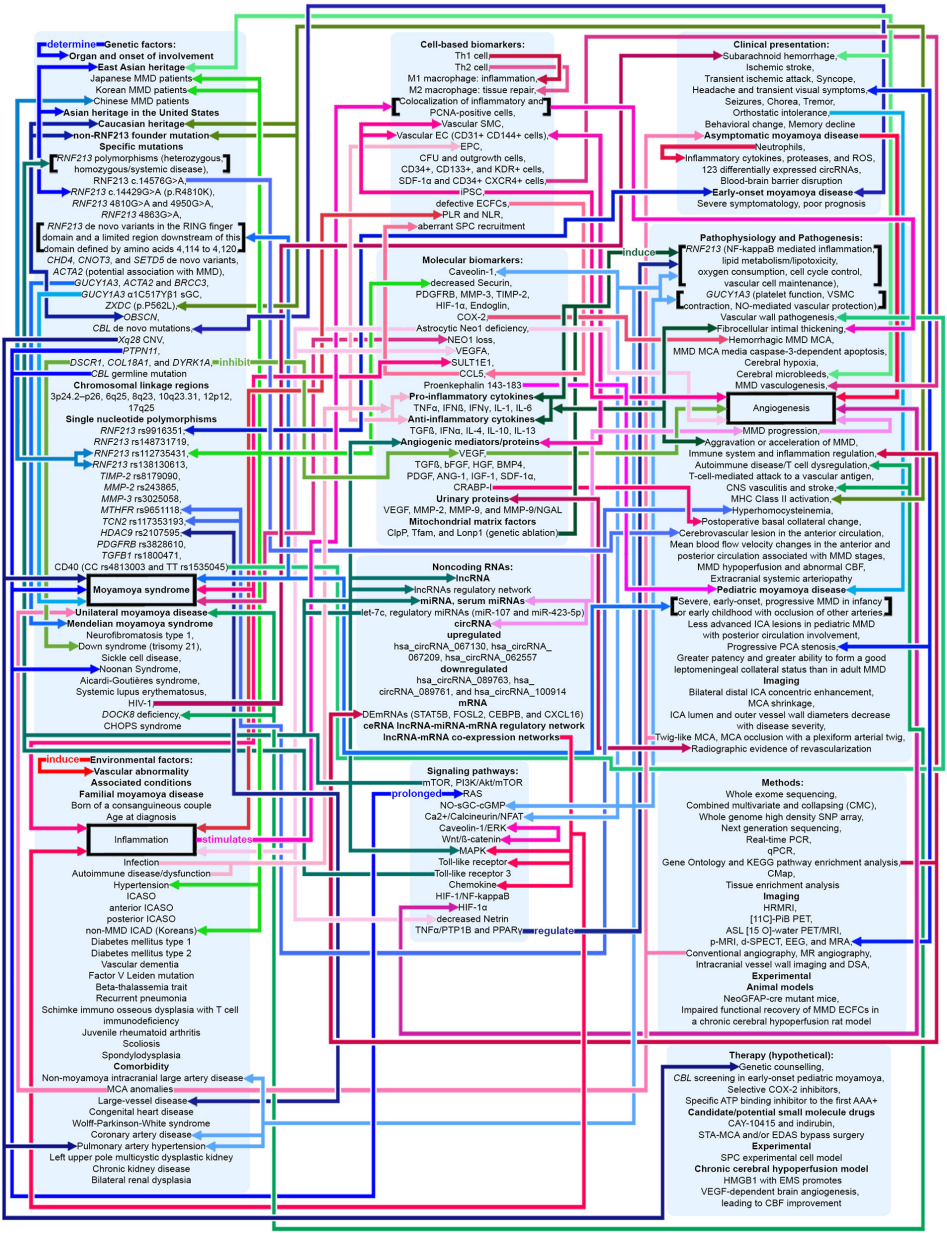
Potential pathophysiologic mechanisms in MMA as related to angiogenesis, inflammation, and genetics. Shades of red indicate that the mechanism relates to angiogenesis and inflammation. Shades of blue indicate that the mechanism relates to genetics. Shades of green indicate that the mechanism relates to both angiogenesis, inflammation, and genetics. Pointed arrows represent stimulatory regulation, double-ended arrows represent bidirectional regulation. See Supplementary material.

The purpose of this review article is to describe the physiological and pathophysiological mechanisms of signaling pathways, cells, and genes relevant to angiogenesis and inflammation in MMA and MMS along with future moyamoya research perspectives and treatment strategies implemented into clinical practice (Figure 4). This article discusses if these mechanisms may be regarded as causative of the angiopathy or if they may be viewed as a consequence of ischemic processes observed in MMA. We also aim to further specify proposed therapeutic and diagnostic targets related to angiogenesis and inflammation in MMA, that may lead to disease-modifying treatment strategies (4, 6, 9, 43–46).

## Methods

References were identified by use of a systematic, comprehensive computerized literature search from January 1, 1983, through July 29, 2022, performed by both authors, using the PubMed, Embase, BIOSIS Previews, CNKI, ISI Web of Science, and Medline databases and the key words “moyamoya,” “angiogenesis,” “anastomotic network,” “moyamoya syndrome,” “molecular mechanism,” “signaling pathway,” “genetics,” “biomarker,” “physiology,” “pathophysiology” “blood-brain barrier,” “endothelial function,” “endothelial progenitor cells,” “intracranial hemorrhage,” “inflammation,” and “stroke” in various combinations. Relevant articles on MMA and supplemental basic science articles almost exclusively published in English were included. References of included publications have been searched for supplementary sources, and 350 publications have consequently been cited in the manuscript. After being reviewed by a member of the panel, the manuscript has been reviewed by five expert peer reviewers. Even though several basic research results about physiologic characteristics of angiogenesis, arteriogenesis, vasculogenesis (13, 14), and associated signaling pathways (15–42) as well as knowledge regarding inflammation in pediatric ischemic stroke (47–56) have been included for the convenience of readers who may be unfamiliar with these topics, this article emphasizes MMA basic, laboratory and clinical research results, future research perspectives, treatment strategies, and their implementation in clinical practice. As several aspects of MMA have been studied in greater detail in comparison to others, distinct topics receive additional attention. Despite substantial progress in the MMA field of research in recent years, the literature in great part remains descriptive. Continued basic and clinical research is essential to further elucidate the pathogenesis of MMA, and to obtain significant results.

## Pathologic characteristics of angiogenesis, inflammation, hemodynamics, vascular wall imaging, vascular regression, and hemorrhage in moyamoya angiopathy

### Angiogenesis, inflammation, and hemodynamics in moyamoya angiopathy

Cerebrovascular diseases may present as a disruption and as aberrations of the intracranial vasculature, including cerebral blood supply (57). Initiation of the pathogenesis of various cerebrovascular diseases has been associated with the vascular wall (57). Stenotic changes in MMA involve the distal intracranial ICA. Disease progression involves the proximal anterior cerebral artery (ACA) (A1), the middle cerebral artery (MCA) (M1), and rarely the posterior circulation (5). MMA vascular wall pathology demonstrates fibrocellular intimal thickening with increased vascular smooth muscle cell (SMC) proliferation, fragmentation and tortuousness of the internal elastic lamina, media attenuation, microaneurysms, and fibrin deposits (5, 58, 59). Thrombosis, a consequence of vessel lumen collapse, may be demonstrated in moyamoya (5, 60). These MMA pathogenetic changes may cause hemorrhagic and ischemic stroke (5). Masuda et al. demonstrated the infiltration of T cells and macrophages into vascular sections without stenosis, indicating that microthrombi may result from chronic inflammation instead of causing this process (5, 61). Presence of microthrombi may not be specific for MMA (5). Inflammation may cause hyperplasia of intimal SMCs and neovascularization through endothelial cell proliferation, leading to lumen stenosis and formation of collaterals (9). In 2006, Takagi et al. demonstrated that apoptosis, evidenced through activated caspase-3, may occur in the MCA media in MMA patients. Consequently, MCA specimens from MMA patients showed vascular wall/medial thinning compared to controls (62). In their 2008 study in 19 adult MMA patients, Kwag et al. suggested that linear and/or non-linear mean blood flow velocity (MBFV) changes in the posterior and anterior cerebral circulation, related to distinct intracranial vessels, may be helpful in both follow-up and initial evaluation of distinct angiographic Suzuki stages of MMA, and may provide results to further ascertain hemodynamic changes related to the disappearance of the bilateral anterior circulation. The research group stated that the MBFV in the ACA, terminal ICA, and the MCA showed a non-linear increase up to Suzuki stage III, and subsequently progressively decreased as far as Suzuki stage VI. Moreover, the ophthalmic artery showed non-linear changes of blood flow velocity, with an MBFV increase as far as Suzuki stage IV, followed by an MBFV decrease as far as Suzuki stage VI. The MBFV of the basilar artery showed a linear increase from a normal velocity at an early MMA stage to a stenotic velocity at a late MMA stage. No statistically significant regression model for the relationship between the angiographic Suzuki stage of MMA and the MBFV in the PCA was evident (63). In their 2011 study in 292 MMA or MMS patients, Lee et al. stated that, in response to superficial temporal artery (STA)–middle cerebral artery (MCA) bypass surgery, flow rates at the vascular anastomosis increased 5 fold to a mean of 22.2 ± 0.8 mL/min. In comparison to adult MMA or MMS patients (23.9 ± 1.0 mL/min; *P* < 0.0001), MCA flow rates were significantly decreased in pediatric MMA or MMS patients (16.2 ± 1.3 mL/min) (64). The research group hypothesized that increased local flow rates may be related to improvement of clinical symptoms. Persistent post-operative complications were low (<5%) (64). Also, the group suggested that eminently increased post-operative MCA flow rates, in comparison to controls, may be related to transient neurologic deficits (28.6 ± 5.6 mL/min; *P* = 0.047), hemorrhage (32.1 ± 10.2 mL/min; *P* = 0.045), and post-operative stroke (31.2 ± 6.8 mL/min; *P* = 0.045) (64). In their 2013 study in 13 MMA patients and 10 healthy, age-matched controls, Chen et al. ascertained the beginning of dynamic cerebral autoregulation impairment at an early MMA stage (65). Every autoregulatory parameter correlated well with the angiographic MMA stage (65). The research group suggested that cerebral autoregulation impairment may progress with MMA progression toward complete vascular occlusion (65). Due to an increased risk of intracranial hemorrhage and ischemia, blood pressure intervention may be warranted (65–67). In 2013, Schubert et al. referred to a characteristic proximal pattern of collaterals (68). In 2015, Baltsavias et al. stated that the previously imprecisely described “moyamoya abnormal network” in pediatric MMA may be specified as a composition of four anastomotic networks with a readily distinguishable vascular structure (69). Accordingly, in their 2015 retrospective study in newly diagnosed 14 pediatric MMA and 11 pediatric MMS patients, Baltsavias et al. described four types of anastomotic networks in pediatric MMA, two deep-parenchymal networks and two superficial-meningeal networks (69). As deep-parenchymal networks the research group detailed the previously undescribed subependymal network and the inner striatal and inner thalamic networks. The subependymal network may be fed by the intraventricular branches of the choroidal system and diencephalic perforators, which, at the level of the periventricular subependymal zone, anastomose with medullary-cortical arteries and also with striatal arteries (69). The inner striatal and thalamic networks may be comprised of intrastriatal connections among striatal arteries and intrathalamic connections among thalamic arteries when MMA compromises the origin of one or additional of their supply sources (69). As superficial-meningeal networks, the research group specified the leptomeningeal and the durocortical networks (69). Apart from the previously described leptomeningeal network observed in the convexial watershed zones, the group described the basal temporo-orbitofrontal leptomeningeal network. The second superficial-meningeal network was detailed as the durocortical network, with a calvarian or a basal location (69). In their 2015 study, Karunanithi et al., using computational fluid dynamics (CFD), evaluated 8 adult hemorrhagic MMA patients treated with encephaloduroarteriosynangiosis (EDAS) revascularization surgery, through analysis of pressure reduction in the right and left ICA before and after EDAS surgery, to ascertain how hemodynamic parameters including pressure reduction and flow rates may be the decisive factor for treatment outcome. The research group stated that pressure drop indicator (PDI), defined as the difference in pressure reduction in the ICA bilaterally, which, by use of patient-specific inflow rates, may be calculated post-operatively and at follow-up, may assist clinicians in reliable risk stratification of MMA patients regarding long-term follow-up (70). Also, PDI may further elucidate the hemodynamic mechanism associated with intracranial hemorrhage in MMA, including recurrent hemorrhage (70). In their 2016 retrospective, 1:2 matched case-control study in 180 MMA patients with or without Type 2 diabetes mellitus (T2DM), Ren et al. suggested that EDAS surgery may be an effective treatment for adult MMA, stating that T2DM patients may gain improvement of symptomatology as well as a more favorable collateral circulation post-operatively. Whereas T2DM was related to a favorable clinical outcome, PCA involvement and late post-operative stroke were identified as predictors of an unfavorable clinical outcome in both study groups (71). In 2016, Story et al. performed a study consisting of a single-institution case series of 204 MMA patients, with an average age at surgery of 9.5 years, who underwent pial synangiosis between 2005 and 2013. Transdural collaterals were present in almost half of all pre-operative arteriograms in MMA patients. These collaterals were demonstrated to be more common in advanced MMA, are associated with stroke as a perioperative complication, and may suggest an increased capacity to produce surgical collaterals post-operatively. Consequently, the research group supports the utility of pre-operative arteriography (72). In their 2016 letter to the editor, Wang et al. stated that, based on their study results, they have established microvascular density as a decisive factor contributing to the result of EDAS, and as a significant predictor of a favorable surgical outcome, potentially assisting to ascertain patients suitable for EDAS. Consequently, in case, during surgery, the cortex appears “white,” the surgical procedure should be direct or combined anastomosis, not EDAS. On the contrary, if an increased number and diameter of vessels are observed which may lead to a “reddish” appearance of the cortex, the patient may be considered suitable for EDAS (73). Regarding their 2017 study results, Qiao et al. stated that blood oxygen level–dependent functional magnetic resonance imaging (BOLD-fMRI) may be an efficient imaging technique to evaluate hemodynamic change in MMA patients (74). In their 2017 comparative study in 41 MMA patients, Qiao et al. suggested that, in comparison to dynamic susceptibility contrast-magnetic resonance imaging (DSC-MRI), multiple inversion time arterial spin labeling (mTI-ASL) may effectively evaluate moyamoya cerebral hemodynamics and assess cerebral ischemia before surgical revascularization and reduction of ischemia after surgical revascularization. The research group indicated that mTI-ASL, not requiring contrast mediums, may be advantageous (75). In their 2018 review article, Yu et al. stated that, in case stenosis or occlusion occurs at the top of the ICA or the first segment of the ACA (A1), the first segment of the MCA (M1) or distal to the anterior choroidal artery (AChA), the AChA can be preserved. The AChA may play a decisive role in MMA (76). In 2019, Fan et al. suggest that their simultaneous hybrid positron emission tomography (PET)/magnetic resonance imaging (MRI) study may support the use of multidelay simultaneously acquired arterial spin labeling (ASL) MRI in clinical evaluation of MMA, in settings where nuclear medicine imaging is not available, and the application of a normative perfusion database to identify aberrant cerebral blood flow (CBF) in MMA patients (77). In 2019, Kronenburg et al. showed that the severity of MMA may be related to the presence of leptomeningeal collaterals and to cerebrovascular reactivity (Figures 1–3) (78). In 2019, Liu et al. proposed a new MMA collateral grading system, reflecting the intracranial collateral circulation status, which correlated well with therapeutic prognosis, hemodynamic status, and severity of symptomatology, which may help evaluate the severity of ischemic and hemorrhagic MMA, ascertain the applicable surgical indication, evaluate the surgical risk, and which may facilitate risk stratification and predict prognosis in MMA (79). In their 2019 retrospective study in 68 adult MMA patients, Zhang et al. showed that direct anastomoses of parasylvian cortical arteries with anterograde hemodynamic sources from the MCA may pose an increased risk of post-operative cerebral hyperperfusion in the course of STA-MCA bypass surgery in adult MMA patients (80). In their 2020 study in 16 MMA patients and 9 atherosclerotic cerebrovascular disease (ACVD) patients, using sodium fluorescein (NaFl) to evaluate blood-brain barrier (BBB) permeability *in vivo* intraoperatively, and using intraoperative indocyanine green (ICG) videoangiography, Lu et al. observed that BBB impairment in MMA may be of increased significance in comparison to ACVD. Regarding their study results, the research group stated that cortical perfusion may be significantly decreased in the cerebral cortex with BBB dysfunction in comparison to a cerebral cortex with an intact BBB in MMA patients. Moreover, the research group suggested that BBB dysfunction may lead to increased cortical perfusion after STA-MCA bypass surgery, subsequently contributing to an increased incidence of post-operative cerebral hyperperfusion syndrome (CHS), contributing to delayed intracranial hemorrhage or transient neurological deterioration in MMA patients (81). In their 2021 10-year follow-up study, Wang et al. determined potential predictors of neoangiogenesis and factors which may influence collateral circulation formation following EDAS (Figure 4) (82). The results of the prospective clinical trial between June 2017 and May 2018 in 106 MMA patients, conducted by Wang et al., suggested that atorvastatin administered at 20 mg per day may be effective and safe for post-operative collateral circulation formation induced by EDAS in MMA patients (45).

### Moyamoya vascular wall imaging, moyamoya vascular regression, and hemorrhagic moyamoya angiopathy

Established perfusion and luminal imaging methods may not provide sufficient image resolution about progression, onset, and differentiation of cerebrovascular diseases (57). Intracranial High-resolution Magnetic Resonance Imaging (HRMRI) of the vascular wall proved to be an effective imaging method regarding evaluation and comprehension and of cerebrovascular diseases (57). Location and pattern of contrast enhancement in intracranial vascular wall imaging may allow novel insight into the etiology of inflammation in cerebrovascular diseases and may have the capability to anticipate treatment and diagnosis (57). Luminal imaging may not be capable of reliably distinguishing between MMS and MMA (57, 83). On vessel wall imaging, MMS, if accompanied by a vasculopathy, e.g., atherosclerosis, may demonstrate outward remodeling and focal eccentric lesion enhancement (57, 83). On the contrary, MMA-infested vascular segments may infrequently enhance without any outward remodeling (57, 83, 84). If MMA-infested vascular segments do enhance, they may show a slightly concentric, homogeneous pattern (57, 83, 84). In 2014, Ryoo et al. performed an HRMRI study in 32 MMA patients and 16 patients with ICAD-related strokes. In addition to evidence of MMA on imaging, MMA patients showed MCA shrinkage and bilateral distal ICA concentric enhancement (85). In 2015, Yuan et al. showed that HRMRI may detect different types of MCA stenosis. On HRMRI, moyamoya MCA segments were depicted through collaterals, homogeneous signal intensity, and concentric stenosis. MCA shrinkage may be associated with MMA progression (86). In their 2016 imaging study, Han et al. suggested that HRMRI may help diagnose intracranial atherosclerosis with increased precision in MMA patients with risk factors for atherosclerosis. A distinct symptomatology of MMA patients without an identifiable atherosclerotic plaque and MMA patients with an identifiable atherosclerotic plaque present may be indicative of distinct pathophysiologic mechanisms and consequently of potentially diverging treatment strategies (87).

In their 2016 retrospective imaging study in 148 consecutive vessel-wall MRI cases, Mossa-Basha et al. stated that vessel-wall MRI of the carotid artery territory may substantially improve differentiation of moyamoya vasculopathies, including MMA, atherosclerotic-MMS, vasculitic-MMS, and steno-occlusive intracranial carotid disease, if combined with traditional imaging techniques (83). In their 2016 study in 20 consecutive MMA patients, using gradient echo T2^*^ weighted imaging (WI) involving high-field MRI, Noshiro et al. suggested that cortical and subcortical vascular hypointensity (CSVH) on T2^*^ WI may be a useful tool for both diagnosis and evaluation of the extent of MMA, demonstrating that MMA revascularization surgery may decrease CSVH (88). In 2017, Qiao et al. showed that cortical thickness in MMA may be multifactorial, including structural reorganization, cerebrovascular accident (CVA) lesions, collateral circulation, and major artery involvement, and may assist as a biological marker to evaluate MMA severity (89). Anomalies of the MCA occur less frequently than anomalies of other major intracranial arteries. MCA fenestration, a duplicated MCA origin, a duplicated MCA, and an accessory MCA may develop due to a fusion failure of the primitive arterial network. Clinically, it may be challenging to differentiate an unfused or twig-like MCA from unilateral MMA, in which stenotic change originates at the MCA. Although MCA anomalies may be asymptomatic, and may not require intervention, knowledge of this configuration of an anomalous MCA may be important in neuro-interventional and neurosurgical practice to perform safe endovascular or surgical interventions. If the twig-like MCA may be identically equal to the persistent fetal network of the primitive MCA remains to be ascertained (90). Regarding their 2019 imaging study results, using intracranial 3.0T vessel wall imaging (VWI) and digital subtraction angiography (DSA), Cogswell et al. suggested a decrease in supraclinoid ICA lumen and outer vessel wall diameters, but no significant change in vessel wall thickness, between 23 North American MMA patients and 23 age-matched controls. Furthermore, the research group showed that outer vessel wall diameters and the ICA lumen may decrease with MMA severity (91). In their 2019 study, using quantitative three-dimensional constructive interference in steady state (3D-CISS) imaging, including 8 hemispheres of 7 MMA patients whose Suzuki angiographic stage had progressed spontaneously during follow-up, Yamamoto et al. demonstrated that, in the course of spontaneous disease progression in early-stage MMA, stages 1–3, the outer diameter of respective arteries may serially decrease in parallel to luminal stenosis. The research group suggested that this mechanism may be associated with MMA pathogenesis (92). In their 2019 quantitative 3D-CISS imaging study, Yamamoto et al. showed that involvement of the P2 segment of the posterior cerebral artery (PCA) in MMA may demonstrate both arterial shrinkage and luminal stenosis. MMA progression in the PCA may additionally promote this vascular wall pathology. The research group hypothesized that, from an embryologic perspective, the pathophysiologic mechanism of MMA pathogenesis may be present in both the PCA and the carotid fork (93).

In their 2014 review article, Wan and Duan stated that hemorrhagic MMA may occur in adult patients of Asian populations, and many factors may contribute to the pathogenesis and the etiology of hemorrhagic MMA. Predominant imaging features of hemorrhagic MMA include aberrant branching and dilatation of the posterior communicating artery (PCoA) or anterior choroidal artery (AChA), as well as multiple microbleeds, potentially prognosticating subsequent intracranial hemorrhage (94). In their 2015 case series of 349 hemorrhagic MMA patients, Wan et al. stated that SAH may be a significant type of hemorrhage in MMA patients, ranking as the fourth most common type after intracerebral hemorrhage (ICH), intraventricular hemorrhage (IVH), and combined ICH and IVH. The research group suggested that SAH may predominantly occur in adult females, and rupture of the transdural anastomosis may be the main cause of this condition (95). In their 2016 Letter to the Editor, Duan et al. stated that revascularization surgery may not have the potential of fully preventing recurrent intracranial hemorrhage. Moreover, the research group stated that their research on the arterial vascular wall, using high-resolution magnetic resonance imaging (HRMRI), has demonstrated that ischemic MMA may have distinct features compared to hemorrhagic MMA, and that all episodes of intracranial hemorrhage may have appeared in MMA patients without plaques (96). In their 2016 case-control study, Liu et al. showed that, in comparison to cerebral hemispheres not affected by intracranial hemorrhage, cerebral hemispheres affected by intracranial hemorrhage may be more susceptible to recurrent intracranial hemorrhage. The study results of the research group demonstrated that dilation of the posterior communicating artery (PCoA) or the anterior choroidal artery (AChA), as well as posterior cerebral artery (PCA) involvement, may be related to initial hemorrhage in hemorrhagic MMA, but not to recurrent episodes of hemorrhage (97). In their 2018 retrospective study in 95 hemorrhagic MMA patients, Wang et al. showed that, through long-term follow-up, EDAS may result in a favorable outcome in hemorrhagic MMA patients. The research group suggested that anterior choroidal artery (AChA)-PCoA dilation may be related to initial intracranial hemorrhage in hemorrhagic MMA, and that recurrent episodes of hemorrhage may be age-related (98). According to their preliminary 2019 cohort study results, Funaki et al. indicated that presence of choroidal collaterals, an anastomosis between the medullary arteries and the posterior or anterior choroidal arteries, may affect the risk of recurrent intracranial hemorrhage in the non-hemorrhagic hemisphere of adult hemorrhagic MMA patients, registered in the Japan Adult Moyamoya (JAM) Trial and assigned to the non-surgical study arm (99). According to their 2019 cohort study results, Funaki et al. hypothesized that choroidal collaterals may be a bleeding spot with an increased risk for recurrent intracranial hemorrhage and a marker of recurrent hemorrhage in hemorrhagic MMA (100). In their 2019 retrospective study, Yu et al. stated that, in comparison to patients with acute idiopathic primary intraventricular hemorrhage (PIVH), patients with acute MMA-associated PIVH may exhibit a lower short-term mortality, be of a younger age, may have a more favorable renal function, and a lower admission blood pressure (101). In their 2020 study, Zhang et al. compared the five-year prognosis in 123 adult hemorrhagic MMA patients who underwent either combined superficial temporal artery to middle cerebral artery (STA-MCA) bypass and EDAS, or EDAS alone. The research group stated that both combined revascularization and EDAS alone may reduce the risk of recurrent hemorrhage in hemorrhagic MMA patients (102). Combined revascularization was found to be superior to EDAS alone regarding the prevention of recurrent hemorrhage (102). In Kaplan–Meier survival analysis, combined revascularization was demonstrated to have a more favorable prognosis compared to EDAS alone, and multivariate regression analysis demonstrated that the combined revascularization procedure may be related to a more favorable outcome (102). In 2021, Wu et al. stated that the choroidal anastomosis may be related to hemorrhagic adult MMA at an advanced stage, suggesting the validation of choroidal anastomosis as an imaging biomarker of hemorrhagic MMA. HRMRI may provide detailed information on both aberrant collaterals and the anatomy in MMA, facilitating risk estimates of moyamoya hemorrhage (Figures 1–3) (103).

## Pathophysiologic characteristics of inflammation in pediatric ischemic stroke

The significance of inflammation in pediatric stroke has become noticeably evident (47). Ischemia may trigger various cascades of inflammatory reactions, both alleviating and aggravating ischemia, including inhibition and activation of inflammation through chemokines, proteases, adhesion molecules, and cytokines (47, 48). Furthermore, it has been demonstrated that the pathophysiology of pediatric stroke may be associated with inflammation (47), as evident in transient cerebral arteriopathy (47, 49) and post-varicella angiopathy (47, 50). Consequently, in pediatric stroke, ischemia may cause inflammation, and inflammation may equally lead to ischemia (47). In comparison to the adult brain, significant differences are evident in the neonatal brain (47). In neonatal stroke, ischemia may be the predominant pathophysiologic mechanism, with inflammation and infection having a significant effect on the degree and course of tissue damage (47). In childhood, ischemia may be caused by an associated inflammatory pathophysiologic mechanism, as evident in MMA, sickle cell anemia, dissection, transient focal arteriopathy, and, increasingly generalized, in generalized vasculitis, meningitides, and genetic arteriopathies such as Deficiency of Adenosine deaminase 2 (DADA2) (47). Focal inflammation is prone to be located in the distal ICA or the proximal medial cerebral artery (MCA), whereas generalized inflammation predominantly affects small arteries (47).

Various genes may be associated with MMA (47, 51). Whether these genes are dysfunctional due to ischemia or inflammation or whether these genes are dysfunctional as such remains to be elucidated (47, 51). The *Ring finger protein 213* (*RNF213*) (17q25.3) genetic variant has been demonstrated to be expressed at an increased level in mature lymphocytes in comparison to lymphoid progenitor cells (47, 51). In MMA, blood levels of circulating endothelial progenitor cells (EPC) may be increased, suggesting that the *RNF213* genetic variant may alter the function of EPCs in the spleen (47, 51). C3, IgG, and IgM have been demonstrated in the vascular wall of MMA patients (47, 52). Fujimura et al. (47, 104) and Young et al. (5, 47) have reviewed signaling cascades and the histology associated with MMA, suggesting an increase in transforming growth factor (TGF), hepatocyte growth factor (HGF), basic fibroblast growth factor (bFGF), and vascular endothelial growth factor (VEGF) in MMA patients (47). These growth factors may be related to angiogenesis and inflammation (47). Extracellular inflammatory biomarkers including matrix metalloproteinase (MMP)-9, interleukin (IL)-8, and prostaglandin may be increased in MMA patients (47). If disease progression may be affected through stimulation or blockade of particular sequences of a signaling cascade remains to be ascertained (47). Blockade of several of such elements may reduce perioperative surgical risk (47, 53).

Cerebral ischemia may initiate an inflammatory signaling cascade causing cell death, which subsequently may initiate inflammation (47, 48). Hypoperfusion may initiate anaerobic glycolysis which may catalyze two main metabolic pathways causing inflammation: sodium-potassium pump failure may cause excitotoxic glutamate release and membrane destabilization. Activation of α-amino-3-hydroxyl-5-methyl-4-isoxazole-propionate and N-methyl-D-aspartate receptors or signaling pathways may lead to both necrosis, and, through intracellular increase of sodium and potassium, to inflammation, oxidative stress, and mitochondrial failure (47, 48). Through blood-brain barrier (BBB) disturbance and membrane degradation, anaerobic glycolysis may lead to inflammation, cyclooxygenase (COX) activation, leukocyte infiltration, and cell adhesion molecule expression. Inflammation may cause both necrosis and apoptosis (47, 48). Inflammation may cause the release of various distinct proteases, chemokines, cytokines, and adhesion molecules which may affect the inflammation process (47, 48). Inflammation may as well be related to coagulation, leading to a procoagulant state through its impact on fibrin formation (47, 48). Also, endothelial inflammation may affect coagulation and lead to blood clot formation (47, 48). The postischemic inflammation pathway in the adult is complex, yet increasingly ascertained (47). An extensive network of anti-inflammatory and pro-inflammatory chemokines, proteases, adhesion molecules, and cytokines exists (47, 48). Initial substrate release predominantly stimulates inflammation within the initial hours and minutes (47, 48). Subsequently, predominantly anti-inflammatory substrates, leading to angiogenesis and recovery, are released (47, 48). Post-ischemic necrotic neurons may release damage associated molecular patterns (DAMPs), thus activating macrophages (47, 48). Macrophages are associated with proinflammatory cytokine release, including IL-1β and TNF-α, which may induce inflammation (47, 48). Also, macrophage IL-23 release may cause T-cell recruitment which, through IL-17 release, may induce inflammation (47, 48). Such an inflammatory reaction may be induced within hours and minutes after ischemia onset (47, 48). Over weeks and days after ischemia, immune cells are associated with anti-inflammatory substance production, such as TGF, insulin-like growth factor (IGF), and IL-20 (47, 48). Purine release may assist in cleaning necrotic cells of debris, and VEGF release may lead to angiogenesis (47, 48). Secretion of anti-inflammatory and inflammatory biomarkers, such as proteases, chemokines, and cytokines, may be ascertainable in the cerebrospinal fluid (CSF) during acute stroke (47, 54, 55). These biomarkers may be related to stroke severity and stroke subtypes, and may further elucidate stroke pathogenesis (47, 54). Various research has been designed for further ascertainment of these distinctive signaling cascades, aiming at modifying factors relating to the post-ischemic disease process (47). Comparison of adult rodents to preterm and/or neonatal rodents demonstrated that, while signaling cascades may be similar, there may be differences between the adult system and the immature prenatal system (47). Various experimental models for age-related ischemia may be warranted to further ascertain these signaling cascades in addition to encourage research into interventions to improve patient outcome (47). The pathophysiology of pediatric stroke may be caused through inflammation, which may exert a specific effect on the inflammatory signaling cascade related to ischemia (47, 56).

## Physiologic characteristics of angiogenesis, arteriogenesis, and vasculogenesis

Moyamoya vessels suggest that aberrant angiogenic, arteriogenic, and vasculogenic processes may be involved in the pathophysiology of the arteriopathy (105).

Physiologic angiogenesis comprises six steps. Step one includes vasodilation, endothelial permeability and periendothelial support. Vasodilation involves NO. VEGF increases vascular permeability and promotes angiogenesis. Angiopoietin (ANG)1 inhibits vascular permeability and stabilizes preexisting vessels. ANG2 removes vascular smooth muscle cells (SMC) and loosens the extracellular matrix (ECM) (13, 14). Matrix metalloproteinases (MMPs) degrade ECM molecules and activate VEGF, basic fibroblast growth factor (bFGF) and IGF-1 (13). Step two includes endothelial cell migration and proliferation. VEGF and VEGFR2 are involved in aberrant, embryonic and neonatal angiogenesis. VEGFR3 is involved in aberrant and embryonic angiogenesis (13). VEGF_120_ initiates angiogenesis. VEGF-C may contribute to aberrant angiogenesis in the adult (13). ANG1 is chemotactic for endothelial cells, potentiates VEGF, and induces angiogenesis (13, 14). bFGF and platelet-derived growth factor (PDGF) affect angiogenesis by attracting inflammatory or mesenchymal cells (13). Markers involved in cell-matrix and/or cell-cell interactions, e.g., αvβ3 integrin, may facilitate endothelial spreading (13). EphrinB2 and platelet endothelial cell adhesion molecule (PECAM)-1 may be associated with aberrant angiogenesis (13). In ischemia, eNOS mediates aberrant VEGF-initiated angiogenesis (106). Step three comprises lumen formation. Endothelial cell thinning or intercalation and fusion of pre-existing vessels may increase vessel diameter and length (13). VEGF121, VEGF165 and their receptors increase lumen formation and vessel length. VEGF189 decreases the luminal diameter, VEGF in combination with ANG1 increases the luminal diameter (13). αvβ3 or α5 integrins influence lumen formation. Thrombospondin (TSP)-1 inhibits lumen formation (13). Step four comprises endothelial survival. Reduced endothelial cell survival causes vascular regression in the embryo (13). Shear stress is essential for vascular maintenance. Endothelial survival factors VEGF, ANG1, and αvβ3 activate p42/44 MAPK, survivin, and PI3K/Akt pathways (13). Step five comprises endothelial differentiation. Specialized endothelial cells are partly determined by their host tissue. Interaction between VEGF and the ECM, causes endothelial cells to become discontinuous and fenestrated (13). Step six comprises remodeling. Vessel replacement by matrix causes vessel branching. A morphogenetic function of VEGFR3, VEGF isoforms, Tie1, vascular cell adhesion molecule-1 (VCAM-1), Jagged, α4 integrin, Gα13 GTP-binding protein, chemokine receptor 4, and fibronectin may be suggested by gene inactivation studies (13). Aberrant angiogenesis is often induced by inflammation. In inflammation and wound healing, VEGF attracts monocytes/macrophages, mast cells, platelets and other leukocytes, which release arteriogenic as well as angiogenic factors, including TGF-β1, bFGF, VEGF, platelet-derived growth factor (PDGF), tumor necrosis factor (TNF)-α, monocyte chemoattractant protein-1 (MCP-1) and IL-8, causing recruitment of endothelial cells, SMCs, platelets, fibroblasts or leukocytes, leading to aberrant angiogenesis (13).

Physiologic arteriogenesis comprises three steps. Regarding step one, in SMC migration and growth, aberrant arteriogenesis causes enlargement of preexisting collaterals after occlusion of the supplying artery. Consequently, endothelial cells express MCP-1 as well as intercellular adhesion molecule 1 (ICAM-1). Vascular wall infiltration and media disruption by monocytes is associated with TNF-α and proteases. Subsequently, endothelial cells upregulate PDGF-BB, bFGF, and TGF-β1, thus inducing SMC growth and vessel enlargement (13). In step two, a lack of fibrillin-1, fibrillin-2, collagen and elastin causes vessel wall weakening and aneurysmal dilatation. Elastin decreases SMC growth, and thereby prevents intimal hyperplasia (13, 107). In atherosclerosis or restenosis SMCs dedifferentiate from a contractile to an embryonic, synthetic phenotype (13). Regarding step three, in remodeling a sustained imbalance between NO and endothelin-1 may induce vasospasms and progress to vascular loss (13).

Physiologic vasculogenesis refers to primitive network formation. VEGF, VEGFR2 and bFGF influence angioblast differentiation. VEGFR1 suppresses hemangioblasts. TGF-β1 and TGF-β receptor 2, α5 integrin and fibronectin affect vasculogenesis (13).

HIF-1β, HIF-1α, and HIF-2α induce angiogenesis and arteriogenesis through VEGFR2, VEGFR1, VEGF, ANG2, PDGF-BB, TGF-β1, Tie1, NOS, IL-8, endothelin-1 and cyclooxygenase (COX)2 expression. Hypoglycemia and a low pH induce vessel growth (13). Vasculogenesis is flow-independent, angiogenesis is flow-dependent and hypoxia regulated (13). bFGF affects vascular tone. NO and P-selectin influence vascular remodeling through shear-stress-induced gene transcription (13).

## Physiologic characteristics of angiogenesis signaling pathways

Signaling pathways associated with a condition may provide an interface of genetic and environmental interaction. Integration and crosstalk between signaling pathways may occur at intracellular nodes where signaling cascades intersect (15), and also at the level of receptor activation (20).

**Figure 5:**
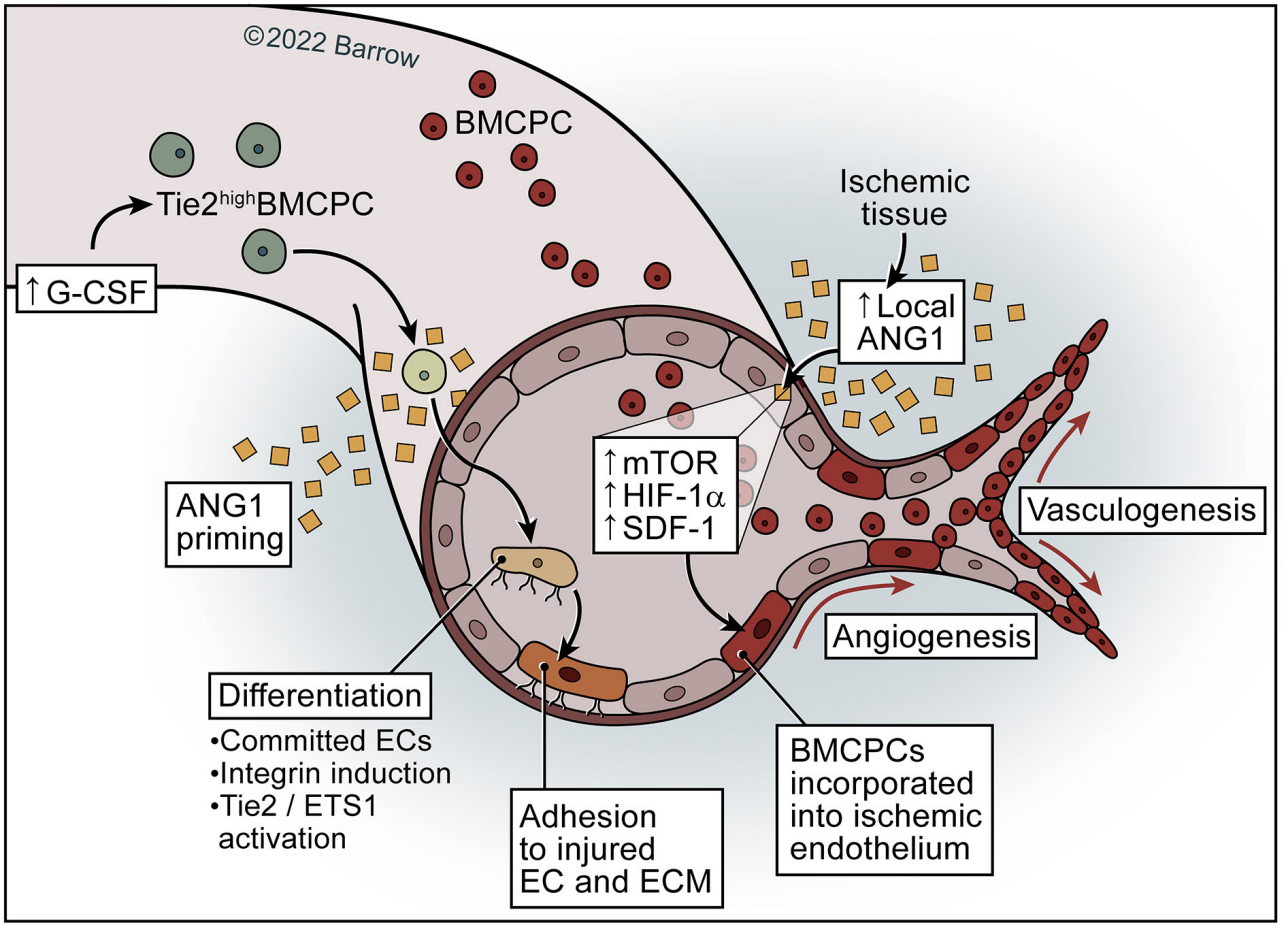
Model of ANG1 in neovascularization. ANG1 may activate and increase mTOR–HIF-1α-SDF-1 signaling in the ischemic endothelium and subsequently facilitate the incorporation of BMCPCs into the ischemic endothelium, leading to neovascularization in ischemic tissue. Priming of mobilized Tie2^high^ BMCPCs with ANG1 causes activation of Tie2/Ets-1, resulting in incorporation of BMCPCs into the endothelial linage and the induction of integrins, which leads to increased adhesion to endothelial cells and the extracellular matrix. See Supplementary material. *Used with permission from Barrow Neurological Institute, Phoenix, Arizona*.

ANG1-Tie receptor tyrosine kinases (Tie)2 binding leads to cross-phosphorylation of cytoplasmic Tie2 tyrosine residues, which recruit adaptor proteins that activate PI3K/Akt, MAPK, Erk, and docking protein 2 (Dok-R) signaling pathways. These pathways are involved in recruiting and sustaining periendothelial support cells (e.g., pericytes, SMCs) that relate to stabilization and maturation of newly formed vessels (18, 19). In quiescent vessels, ANG1 recruits Tie2 to cell-cell contacts, forming complexes with Tie2 from adjoining cells, thus activating PI3K/Akt signaling (16, 18, 19, 23). Migrating endothelial cells cause ANG1 to recruit Tie2 into contact with the ECM, which causes the formation of focal adhesion complexes and activation of PTK2/FAK, MAPK-1/ERK2, and MAPK3/ERK1; this, in turn, causes sprouting angiogenesis (16–22). Activation of Tie1-Tie2 heterodimers depends on β1 integrin (21). In ischemia, ANG1 causes synchronous activation of Tie2 and integrin signaling, which is related to angiogenic remodeling and tightening of endothelial cell junctions (Figure 5) (18). Tie1 deficiency impairs ANG1-induced Tie2 and Akt phosphorylation and FOXO1 inactivation, leading to FOXO1 nuclear translocation (21). Inflammation causes cleavage of the Tie1 ectodomain, which results in a switch of ANG2 from a Tie2 agonist to a Tie2 antagonist, linked to a positive feedback loop of FOXO1-driven ANG2 expression, causing endothelial cell destabilization *via* β1 integrin, vascular remodeling, and leakage (21, 24, 25). Autocrine secretion of ANG2 disrupts connections between endothelium and perivascular cells, causing cell death and vascular regression (16) that lead to impaired barrier properties of brain endothelial cells and intracranial hemorrhage and ischemia (16, 26). ANG2 and VEGF block ANG1-mediated stabilization and maturation, resulting in endothelial cell migration and proliferation and, then, angiogenic neovascularization (18). ANG1-Tie2 activation stimulates recruitment of ABIN-2 that, in turn, creates suppression of NF-κB, a pro-inflammatory transcription factor, and protection of endothelial cells from apoptosis. Truncated ABIN-2 inhibits ANG1 from preventing endothelial cell death (27).

The erythropoietin (Epo)/Epo receptor (EpoR) signaling pathway induces proliferation, migration, chemotaxis, and angiogenesis, and inhibits apoptosis (28, 29). EPO signaling potentially inhibits apoptotic pathways triggered by ischemia and may reduce hypoxic injury by promoting or facilitating angiogenesis (28). Cytokines and growth factors associated with hematopoiesis may also be involved in angiogenesis (31). Endothelial cells expressing EPO-R respond to EPO by differentiation into vascular structures, associated with JAK2 phosphorylation, cell proliferation, and MMP-2 production (30, 31).

The erythropoietin-producing human hepatocellular receptor (Eph)/Eph receptor-interacting protein (ephrin) signaling pathway is involved in vasculogenesis and tissue homeostasis (32, 35). Eph-ephrin bidirectional signaling affects both receptor- and ephrin-expressing cells and segregates Eph-expressing cells from ephrin-expressing cells (33–35). Eph and ephrin may contribute to vascular development by restricting arterial and venous endothelial mixing thus stimulating the production of capillary sprouts and also by stimulating mesenchymal differentiation into perivascular support cells (32). EphA receptor activation may be involved in VEGF-induced angiogenesis (32). Cooperation between ephrin-A1 and Slit2 regulates a balance between pro- and antiangiogenic functions of Slit2, suggesting Slit2 may differentially regulate angiogenesis in the context of ephrin-A1 (36). The Eph family transmembrane ligand ephrin-B2 marks arterial but not venous endothelial cells. The ephrin-B2 receptor Eph-B4 marks veins but not arteries. Differences between arteries and veins may be in part genetically determined, suggesting that reciprocal signaling between arterial and venous endothelial cells is essential for morphogenesis of the capillary beds (37). Interaction of ephrin-B2 on arterial endothelial cells and Eph-B3 and Eph-B4 on venous endothelial cells may define the boundary between arterial and venous domains (37). EphB2 and ephrin-B2 expression on mesenchymal cells suggests involvement in vessel wall development *via* endothelial-mesenchymal interaction (38). Absent ephrin-B2 expression in mice disrupts embryonic development of the vasculature due to a deficient restructuring of the primary network (39).

The Janus kinase-signal transducer and activator of transcription protein (JAK-STAT) signaling pathway includes cytoplasmic signal transducer and activator of transcription (STAT)1, STAT2, STAT3, STAT4, STAT5a, STAT5b, and STAT6. STATs are activated by tyrosine phosphorylation in response to external stimuli, including cytokines, growth factors, and hormones. Ischemia leads to estradiol-, IL-6-, EPO-, and G-CSF-mediated tyrosine phosphorylation and activation of STAT3. STAT3 dimerization and translocation to the nucleus stimulate binding of DNA regions in STAT-inducible elements, which leads to transcription of neuroprotective genes linked to estradiol-mediated neuroprotection and neuronal survival. Endothelial STAT3 activation causes endothelial cell migration and proliferation, leading to angiogenesis and ECM remodeling that are important in long-term post-stroke recovery (40–42).

## Moyamoya angiopathy related angiogenesis and inflammation signaling pathways

The pathogenesis of MMA and MMS may be associated with infection and inflammation (5, 108). Imbalance of angiogenic and vasculogenic mechanisms has been suggested to be a potential cause of MMA (105). Aberrant expression of angiogenic factors, adhesion molecules, and mitogens, and/or an aberration of the cellular immune response to cytokines and growth factors may indicate an association of hematopoietic as well as inflammatory signaling pathways with cells of the vasculature, which has been hypothesized to constitute an essential pathophysiologic mechanism in MMA pathogenesis (Figure 4) (5, 109–113).

Phosphatidylinositol 3-kinase (PI3K)/protein kinase B (Akt)/mammalian target of rapamycin (mTOR) signaling pathway activation may occur due to Ras mutation, loss of phosphatase and tensin homolog, or increased expression of growth factor receptors. The PI3K signaling pathway is involved in blood vessel formation during embryogenesis. Embryos with a kinase-dead PI3K p110α catalytic subunit develop vascular defects. The PI3K/Akt signaling pathway modulates expression of angiogenic factors (e.g., NO, ANGs) (114). Activated Tie2 stimulates the PI3K signaling pathway, which activates the protein kinase B/Akt, eNOS, MAPK, and docking protein 2 (Dok-R)/cytoplasmic protein NCK1 (Nck)/p21-activating kinase (Pak) signaling pathways. These pathways affect endothelial cell survival and NO synthesis (115). VEGF stimulation of endothelial cells activates the PI3K pathway and leads to cell migration. Endothelial activation of Akt1 is associated with structurally abnormal blood vessels. PI3K/Akt/mTOR pathway inhibition decreases VEGF and angiogenesis (114). Increases in caveolin-1 lead to decreases in ceramide synthesis, inhibiting the Akt signaling pathway, cell proliferation, migration, and invasion, thus inhibiting PI3K activity (116). Hypoxia-inducible factor (HIF)-1 expression is associated with the PI3K/Akt signaling pathway in MMA (9, 117). The PI3K/Akt signaling pathway in endothelial cells may lead to transcriptional activation of *Ring finger protein 213* (*RNF213*) (17q25.3). Inhibition of the PI3K/Akt signaling pathway has been demonstrated to decrease inflammation in autoimmune diseases (9, 118). MMA, if associated with inflammation, may be related to the PI3K/Akt pathway (9). Molecular networks may associate sGC with E3 ubiquitin-protein ligase RNF213 (RNF213) (119). Nuclear factor of activated T-cells 1 (NFAT1), a RNF213 ubiquitin target downstream of the non-canonical Wnt/Ca2^+^ signaling pathway, is a pivotal molecule (119, 120). Calcineurin/NFAT signaling activation through VEGF in human EPCs may lead to increased expression of Nitric oxide synthase, endothelial (eNOS) and generation of NO (119, 121). NFAT may regulate sGC expression by NFAT1 binding to the Guanylate cyclase soluble subunit alpha-1 (GUCY1A3) consensus sequence (119, 122). PI3K/AKT activation may cause glycogen synthase kinase (GSK)-3β inactivation, leading to proteasomal degradation of NFAT1 (119, 123). The impact on NFAT1 by PI3K/AKT might be mediated through RNF213, as PI3K/AKT may be an upstream regulator of *RNF213* expression in endothelial cells (119, 124). Moreover, *Nuclear Factor Of Activated T Cells 1* (*NFAT1*) (18q23) upregulation may be mediated through RNF213 S-nitrosylation (119, 125). S-nitrosylation means posttranslational modification through addition of a nitrosyl group to the reactive thiol group of cysteine, forming S-nitrosothiol, which constitutes a pivotal mechanism in NO-mediated signal transfer (119). Ubiquitin ligase S-nitrosylation may lead to its auto-ubiquitination, thus increasing its substrate levels. NFAT, through cGMP-dependent protein kinase (PKG) activation, which leads to GSK-3β phosphorylation, may be regulated through sGC (119, 126). Furthermore, caveolin may be associated with NO signaling regulation (119).

Caveolin-1, an ~ 21–24 kDa integral membrane protein, is present predominantly in plasma membrane caveolae, 50–100-nm flask-shaped invaginations, where it functions as a scaffold to arrange a multitude of molecular complexes which regulate diverse cellular functions. Caveolin-1 may be regulated through the Ca2^+^/calcineurin/NFAT signaling cascade (119, 127). Caveolin-1 has been stated to be related to pulmonary arterial hypertension, coronary artery disease, and MMA (119). Caveolin-1 functioning may be sufficiently studied in pulmonary arterial hypertension (119, 128–130). In comparison to healthy controls or cerebral atherosclerotic stroke patients, caveolin-1 levels have been demonstrated to be decreased in MMA, and were shown to be distinctly decreased in patients with the *RNF213* R4810K genetic variant (119, 131). If RNF213 bears an indirect or a direct relation to caveolin-1, remains to be ascertained, e.g., caveolin-1 may be a target object for ubiquitination through RNF213. eNOS release is related to caveolin-1. eNOS release produces NO, which may be metabolized through sGC. eNOS binding to the caveolin-1 scaffolding domain has been associated with eNOS inactivation (119, 132). Caveolin-1 absence may lead to dysfunction of eNOS, which has been related to cerebrovascular diseases (119). NF-κB and HIF-1 are involved in inflammation regulation (9, 133). *RNF213* genetic variants may cause NF-κB-associated inflammation, leading to VSMC damage, which is characteristic of MMA pathophysiology (119). RNF213 may lead to lipotoxicity-mediated protection of cells against inflammation and endoplasmic reticulum (ER) stress (119, 134). RNF213 depletion may cause NF-κB pathway inhibition during exposure to palmitate, may reestablish the cellular lipidome, and may stabilize the expression of the ER stress gene (119, 134). Recent research has demonstrated that RNF213 may concur with Ubc13, the E2 enzyme, leading to K63-linked polyubiquitin chain generation (119, 135, 136). K63 linkages have been associated with protein sorting, removal of defective mitochondria, innate immune responses, DNA repair, and with regulation of NF-κB transcription factor activation (119, 137). Deletion of Lys-63-specific deubiquitinase BRCC36 (BRCC3), an E3 ligase cleaving K63-linked polyubiquitin chains specifically, has been related to X-linked MMS (119, 138). BRCC3 may be associated with DNA damage response, and may regulate an abundance of such polyubiquitin chains in chromatin (119). The majority of genetic changes in the *RNF213* RING finger domain proven in MMA patients may diminish E3 ligase activity, and various of these genetic changes may induce NF-κB activation (119, 136). Such genetic changes, which may lead to NF-κB activation, may include both Caucasian cysteine/histidine mutations and proline mutations, such as P4033L in a Caucasian patient as well as p.P4007R in a Chinese patient (119). In association with NF-κB, these genetic changes may lead to apoptosis (119). The p.D4013N genetic variant may neither affect NF-κB activation nor E3 ligase activity (119). Point mutations in both the Walker B and A motif of the AAA domains, may fully eliminate NF-κB activation through genetic changes in the *RNF213* RING finger domain (119). Consequently, NF-κB signaling pathway-mediated inflammation may be suppressed in absence of *RNF213*, while inflammation may be augmented through *RNF213* genetic variants in MMA patients (119). With respect to NF-κB activation, *RNF213* genetic variants may be associated with gain of function (119). RNF213 may as well regulate immune cell maturation and differentiation, the cell cycle, and mitochondrial function. These characteristics may be associated with MMA pathogenesis (119). Caveolin-1 is related to inflammation, and may be associated with MMA (9, 139, 140). Caveolin-1 serum levels were shown to be decreased in MMA, and demonstrated to be significantly decreased in MMA patients with the *RNF213* genetic variant (9, 131). Caveolin-1 has been associated with angiogenesis (9, 141, 142), along with a bidirectional interaction between the Caveolin-1/ERK and the Wnt/β-catenin pathway (9, 143).

In 2000, Galbiati et al. hypothesized that caveolin-1 expression may control Wnt/β-catenin/Lef-1 signaling through modulating the intracellular β-catenin localization (144). The Wnt signaling pathway may be related to angiogenesis (9). In 2016, Scholz et al. demonstrated that the endothelial RSPO3-driven non-canonical Wnt/Ca2^+^/NFAT signaling pathway may be associated with vascular stability maintenance, providing insight into vascular remodeling mechanisms (120). Furthermore, the research group stated that *RNF213* in vascular endothelial cells may be associated with the Wnt signaling pathway and angiogenesis regulation (9, 120). Under physiologic conditions, stimulation of endothelial cells through shear stress or growth factors may induce the Ca2^+^-calmodulin signaling cascade (119). Calmodulin may accelerate dissociation of eNOS from caveolin-1 and may enhance eNOS generation through Calcineurin/NFAT1 (119) RNF213, which may degrade NFAT1 by means of the ubiquitin proteasome system, may not be activated in absence of a pathological stimulus (119). From L-Arginine, eNOS subsequently generates NO, which may diffuse into vascular smooth muscle cells (VSMCs) (119). In VSMCs, NO may induce sGC to generate cGMP. cGMP may subsequently activate the cGMP-dependent protein kinase (PKG), leading to VSMC relaxation (119). Under pathological conditions, in which a viral infection may lead to destruction of mitochondria, *RNF213* may be up-regulated and may inhibit the generation of eNOS through NFAT1 degradation (119). *RNF213* genetic variants in MMA patients may sustain an inflammation even after remission of the causative infection, which may lead to sustained impairment of the cGMP signaling pathway (119). *GUCY1A3* genetic variants may cause an identical environment (119). cGMP signaling pathway impairment may lead to endothelial dysfunction, fibrosis, impaired vasodilation, and proliferation of VSMCs (119). Such processes may lead to intimal hyperplasia with fibrous thickening, which has been associated with MMA pathogenesis (119). As cGMP signaling pathway impairment may lead to endothelial-to-mesenchymal transition and dedifferentiation of VSMCs, those cells may cause fibrosis (119). Lessened protection against stroke may constitute a pathophysiologic mechanism of MMA arterial stenosis (119). RNF213 may possess antibacterial and antiviral properties and may regulate lipotoxicity, whereas sGC may provide protection against homocysteine (119). Bacterial and viral infection may cause mitochondrial dysfunction and interferon (IFN) I generation, which may lead to increased *RNF213* expression (119). In case genetic variants in MMA patients cause a dysfunction of sGC or RNF213, vascular damage related to infection, dyslipidemia, and homocysteine may lead to chronic inflammation (119). Moreover, *RNF213* genetic variants may cause inflammation through NF-κB, leading to VSMC damage, a characteristic of MMA pathogenesis (119). In their 2016 study in 15 adult MMA patients, Gao et al. stated that expression patterns of long non-coding ribonucleic acids (lncRNAs) may differ between MMA patients and healthy controls (10). Various signaling pathways related to smooth muscle contraction, vasculogenesis, and immune response may be associated with the regulatory mechanism of lncRNAs (10). Mitogen-activated protein kinase (MAPK) signaling pathway was found to have a central function in this regulatory network of signaling pathways (10). In 2021, Sarkar and Thirumurugan demonstrated the regulation of *RNF213* through the TNFα/PTP1B signaling pathway and PPARγ, suggesting that *RNF213*, similar to TNFα, may constitute an additional connection between MMA, inflammation, insulin resistance, and obesity (11). Toll-like receptors (TLR) have been ascertained to be essential in activating the innate immunity through recognition of distinct patterns of microbial constituents. Toll-interleukin-1 receptor (TIR) homology domain-containing adapter protein Myeloid differentiation primary response 88 (MYD88) may be indispensable for the induction of pro-inflammatory cytokines induced by all TLRs (145). In 2020, Key et al. stated that in MMA, the low penetrance of *RNF213* mutations may be modified through dysfunctions in the TLR3 signaling pathway or the mitochondria (Figure 4) (146).

Due to infections or autoimmune diseases and induced by inflammatory cytokines, every signal transduction pathway involved in MMA may be reciprocally activated by *RNF213* (9).

## Moyamoya angiopathy cell-based biomarkers

Derived from the bone marrow, circulating endothelial progenitor cells (EPC) are involved in postnatal physiological and pathological neovascularization (9, 147, 148). Circulating EPCs have become objects of moyamoya research, referring to the hypothesis that MMA is associated with constant vascular remodeling, involving both the subsequent angiogenesis from collateral development as well as the primary arteriopathy. SMC proliferation in the vascular wall of affected arteries has frequently been demonstrated in MMA (2, 61). Analysis of smooth muscle progenitor cells (SPCs) isolated from the blood of MMA patients demonstrated a differential expression exceeding 200 genes, including a decreased CD31 expression, and irregular tube formation in assays in comparison to matched controls (2, 149). Studies have indicated the migration of endothelial cells into the ICA intima in stenotic sections in moyamoya, hypothesizing that these cells might be involved in both distal collateral development and proximal arterial narrowing (2, 150). CD34+ cells, a subpopulation of endothelial progenitor cells, have been reported to be increased in the blood of MMA patients compared to healthy controls and also when compared to patients with non-MMA intracranial arterial stenosis (2, 151, 152). Inconsistent results have been obtained from research into CD34+ cells in pediatric MMA. Kim et al. performed a study in 28 pediatric MMA patients, demonstrating decreased levels as well as a defective function of CD34+ cells compared to 12 healthy volunteers (2, 9, 153). Rafat et al. performed a study in 20 adult MMA patients, demonstrating an enhancement of circulating EPCs. The research group suggested an involvement of circulating EPCs in angiogenesis and arteriogenesis in MMA (9, 154). A decrease in EPCs following revascularization surgery in MMA has also been reported (9, 155). EPCs secrete angiogenetic factors including ANG1, hepatocyte growth factor (HGF), VEGF, stromal-derived factor-1*a* (SDF-1*a*), bFGF, PDGF, and IGF-1 (9, 152, 154, 156–158). Tinelli et al. morphologically, phenotypically, and functionally characterized circulating EPCs from the peripheral blood of a homogeneous group of adult Caucasian, non-operated MMA patients and healthy controls, suggesting that a significantly reduced circulating EPC level may be a potential marker of MMA (105). Analyzing the function of circulating EPCs *in vitro*, as measured by assays of colony formation and tube formation, may indicate a significantly decreased function of these cells in MMA (2, 9, 159). Choi et al. suggested an impaired functional recovery of EPCs *in vivo* in moyamoya patients in comparison to controls (9, 160). In 2008, Jung et al. stated that distinct characteristics of circulating EPCs (CFU numbers and tube formation were found to be lower in advanced MMA cases than in those with early stage disease, and outgrowth cells were more frequently detected in those with early MMA and moyamoya vessels than in those with advanced MMA) may reflect mixed conditions of aberrant vasculogenesis and vasculars occlusion in MMA pathogenesis (159). Regarding their 2008 study results, Yoshihara et al. suggested that an increased level of CD34+ cells, related to ischemia, may be correlated with neovascularization of the human arterial cerebral circulation at sites of ischemic brain injury (151). In their 2010 study, Kim et al. demonstrated that pediatric MMA may be related to reduced expression of circulating EPCs, to proneness to senescence, defective tube formation, and impaired differentiation. Such a limited capacity of EPCs may lead to insufficient cerebrovascular repair or aberrant vessel formation (153). In their 2011 study, Ni et al. suggested that binding of CXCR4 on CD34+ cells to mediate CD34+ cell migration may lead to an increased level of SDF-1α, hypothesizing that increased levels of circulating SDF-1α and CD34+ CXCR4+ cells in MMA patients may be associated with moyamoya vasculogenesis (152). In their 2011 study, using supraclinoid ICA specimens from two adult MMA patients, Sugiyama et al. stated that VEGFR2- and CD34-positive cells were abundantly demonstrated in the thickened intima of occlusive arterial lesions, clustered especially in the superficial layer of the thickened intima. Also, the research group demonstrated that CD34-positive cells expressed von Willebrand factor on the surface of the thickened intima and were positive for α-smooth muscle actin in the deeper layer, suggesting that circulating EPCs may be associated with occlusive arterial lesion development in MMA (150). In their 2018 study, Bao et al. researched circulating endothelial cells (CECs) in the plasma of 66 MMA patients compared to 81 healthy controls, showing that the amount of CECs was negatively correlated with concomitant disorders including coronary heart disease, diabetes mellitus, and hypertension in MMA patients (161). In their 2021 study, Matsuo et al. demonstrated that vulnerability to shear stress, caused through an aberrant peri-endothelial matrix, may be a predominant characteristic of MMA (162). The research group stated that the peri-endothelial extracellular matrix may be important regarding endothelial protection, cell adhesion and migration, and that an altered peri-endothelial matrix in MMA may contribute to endothelial vulnerability to vascular wall shear stress. Invading EPCs, which repair endothelial damage, may produce excessive hyaluronan and chondroitin sulfate in the intima, and may lead to vascular stenosis (162). In 2021, Wang et al. stated that collateral vessel formation in encephaloduroarteriosynangiosis (EDAS) surgery, a common method for indirect revascularization, may be associated with angiogenesis, and that the EPC count may be essential for facilitating collateral circulation formation. The research group hypothesized that EDAS may prove particularly advantageous for severe ischemic or younger MMA patients (163). In their 2021 study, Wang et al. performed comprehensive profiling of the protein profiles expressed in serum-derived exosomes (SDEs) of MMA patients performing Tandem Mass Tag-labeled quantitative proteomics, demonstrating disturbed actin dynamics in MMA patients, with actin-related protein 2/3 (ACTR2/3) and Cofilin-1 (CFL1) downregulation in SDEs. Distinct expression of immune-related proteins was shown in exosomes, suggesting an alteration of immune responses in hemorrhagic MMA patients. Also, the research group stated that exosomes in hemorrhagic MMA may lead to vascular endothelial cell (EC) proliferation, potentially by induction of mitochondrial dysfunction by means of oxidative phosphorylation and an aberrant electron transport chain (164).

In their 2022 review article, Xue et al. reviewed recent progress and pitfalls in MMA induced pluripotent stem cell (iPSC) research, providing a perspective of iPSC molecular mechanisms and novel MMA treatment strategies (165). In their 2016 study, Hamauchi et al. demonstrated that downregulation of ECM receptor-related genes may be related to impaired angiogenesis in iPSC-derived ECs of MMA patients. The research group stated that upregulation of splicing regulation-related proteins may imply varieties of splicing patterns between ECs of MMA patients and controls (166). In 2016, Cardano et al. described the establishment of an induced pluripotent stem cell (iPSC) line from an 8-year-old female patient with ischemic MMA (167). In 2016, Cardano et al. described the establishment of an induced pluripotent stem cell (iPSC) line from a 55-year-old male patient with hemorrhagic MMA (168). In 2020, Tokairin et al. performed a study in 3 MMA patients and 3 independent healthy controls, which determined vascular smooth muscle cells (VSMCs) from neural crest stem cells using patient-derived induced pluripotent stem cell (iPSC) lines to detail the transcriptome profile and the biological function of MMA VSMCs, suggesting that MMA pathology may be influenced by naive endothelial cells (EC), whereas MMA VSMCs may require specific environmental factors, thereby further elucidating MMA pathophysiology. The research group stated that, in addition to the existing iPSC derived EC model, their iPSC-derived VSMC model may further ascertain therapeutic and diagnostic objectives in MMA (169). In their 2021 study, Mao et al. demonstrated the generation of an induced pluripotent stem cell (iPSC) line HUSTTJi001-A from an MMA patient with a *RNF213* genetic variant. The research group stated that this iPSC line may show pluripotent biomarkers, may have the potential for *in vitro* differentiation into three germ layers, may be suitable for ascertainment of MMA cellular mechanisms, for the selection of therapeutic targets, and for drug development (170).

In 1993, Masuda et al. performed an autopsy study in 6 MMA patients, using immunohistochemical staining by cell-type-specific monoclonal antibodies, stating that SMCs in MMA may be proliferating in occlusive lesions of intracranial major arteries. Furthermore, the research group stated that colocalization of proliferating cell nuclear antigen (PCNA)-positive and inflammatory cells, including T cells and macrophages, may suggest that inflammation could induce proliferation of SMCs and thus contribute to formation of intracranial occlusive lesions in MMA (61). In their 1993 case report, Panegyres et al. suggested that the pathogenesis of unilateral MMA, associated with stroke and terminal ICA occlusion, subsequent to proliferation of subendothelial fibrous tissue and infiltration of mononuclear cells, T cells, into the carotid vascular wall, may be related to a T-cell-mediated response to a vascular antigen (171). Also, the research group stated that animal experiments on dogs may maintain this hypothesis (171, 172). In their 2014 study in 25 MMA patients and 22 healthy controls, Kang et al. showed that, through a suitable cell culture condition, circulating smooth-muscle progenitor cells (SPCs) may be established from the peripheral blood of MMA patients. In comparison to controls, SPCs obtained from MMA patients may demonstrate characteristic differentially expressed genes (DEGs) associated with vascular development, immune response, cell migration, and cell adhesion (149). The 2019 *in vivo* study results obtained by Choi et al. demonstrated impaired functional recovery of MMA endothelial colony-forming cells (ECFCs) in a chronic cerebral hypoperfusion rat model, in comparison to normal control ECFCs, which showed decreased apoptosis as well as increased neurogenesis and vasculogenesis, suggesting an involvement of ECFCs in MMA pathogenesis (160). In 2021, Ma et al. suggested a positive correlation between neutrophil-to-lymphocyte ratio (NLR) and platelet-to-lymphocyte ratio (PLR) biomarkers in MMA patients, which may further elucidate the pathology of inflammation in MMA pathogenesis (Figure 4) (173).

## Moyamoya angiopathy molecular biomarkers

Treatment of an underlying inflammatory disease may lead to remission of MMA symptomatology (9). An immune response related to angiogenesis may be facilitated through M2 macrophages which may be induced through anti-inflammatory cytokines such as TGF-β, interferon (IFN)-α, IL-13, IL-10, and IL-4. Fujimura et al. demonstrated that CXCL5 and CD163 serum levels of MMA patients were significantly increased compared to controls, hypothesizing that MMA pathophysiology may be related to M2 macrophages (9, 174). Anti-inflammatory cytokines may induce angiogenetic markers. TGF-β of Treg/Th17 cells with distinct CD4+ T-helper cell subsets has been demonstrated to be associated with aberrant angiogenesis in MMA by means of VEGF signaling regulation (9, 175). Increase in angiogenetic markers including VEGF, HGF, PDGF, bFGF, cellular retinoic acid-binding protein-1 (CRABP-1), HIF-1 and MMPs may be associated with MMA (Figure 4) (9, 109, 154, 176–185). These markers have been hypothesized to be associated with proliferation of the intima as well as angiogenesis through their influence on endothelial cells, and with progression or initiation of MMA (9). Pro-inflammatory cytokines, including IL-6, IL-1, TNF-α, IFN-γ, and IFN-β, which may induce the pro-inflammatory, *RNF213*-dependent cytokine pathway, may have a different mechanism of action compared to cytokines involved in anti-inflammatory cytokine pathways (9). In 2016, Bang et al. demonstrated a correlation between caveolin-1, *RNF213*, and endothelial function in MMA (131). Also, caveolin-1 may be associated with negative arterial remodeling in MMA (186). VEGF levels have been demonstrated to be associated with caveolin-1 levels but not with MMA, suggesting that a change in plasma VEGF levels may not be a primary cause of MMA (131). In 2013, Hitomi et al. observed that iPSC-derived vascular endothelial cells (iPSECs) from MMA patients showed impaired angiogenic function. The *RNF213* R4810K genetic variant may become clinically manifest as aberrant angiogenesis through downregulation of Securin expression. The resulting defects in angiogenesis are considered MMA risk factors (187). In their 2013 high-throughput analysis of MMA autoantibodies, Sigdel et al. ascertained 6 MMA-related autoantibodies against EDIL3, ROR1, CTNNB1, STRA13, GPS1, and APP, providing important insight into the immune-mediated pathogenesis of MMA, and potentially advancing diagnostic tools for use in clinical practice (46). Accordingly, MMA-related autoantibodies against APP may be associated with an increased risk for hemorrhagic stroke (46, 96). In 2014, Jeon et al. demonstrated that increased CSF CRABP-I levels may be related to bilateral adult MMA. Also, the research group suggested that post-operative basal collateral vessel decrease could be associated with CRABP-I expression levels (188). In 2016, Zhang et al. demonstrated that COX-2 was up-regulated in the MMA MCA, predominantly in hemorrhagic MMA patients, hypothesizing that COX-2 may be associated with MMA pathogenesis, and potentially with hemorrhagic stroke in MMA (189). Based on their 2017 study result, Phi et al. hypothesized that in MMA, defective ECFCs may lead to aberrant recruitment of SPCs toward critical locations in the vasculature by means of chemokine (C-C motif) ligand 5 (CCL5) (190). In their 2018 prospective study in 11 MMA patients, Ishii et al. observed changes in biomarkers associated with tight junctions in the blood-brain barrier (BBB). The research group stated that their preliminary results may indicate that significant hemodynamic change and transient neurologic symptoms (TNS) in some patients may be related to BBB disruption after direct MMA bypass surgery (191). In 2018, Yokoyama et al. demonstrated that CSF proenkephalin 143–183 may be a useful diagnostic biomarker in pediatric MMA. The effect of enkephalin peptides by means of delta opioid receptor or opioid growth factor receptor may be related to MMA pathophysiology, suggesting an association between temporal changes in moyamoya collateral vessels and concentration of proenkephalin (192). In 2020, Surmak et al. showed that a [^11^C]-PiB PET signal related to intracranial inflammation in MMA patients and a single relapsing-remitting multiple sclerosis (RRMS) patient may be corresponding to functional cerebral imaging of SULT1E1, suggesting that significant focal [^11^C]-PiB PET signals may be received from the inflamed living human brain (193). In 2021, Han et al. suggested that elevated CSF and serum sortilin levels may be associated with MMA onset, and, in addition to levels of proinflammatory cytokines, may be effective markers in clinical practice. The research group hypothesized, that sortilin may break through a compromised blood brain barrier (BBB), may consecutively induce inflammation, and thus induce MMA (194). In their 2021 study, Ren et al. demonstrated that cortical astrocytic neogenin (NEO1) deficiency may be associated with MMA pathogenesis. NEO1, *a member of deleted in colorectal cancer* (*DCC*) *family netrin receptors*, was reduced in brain specimens of MMA patients. Astrocytic Neo1-loss resulted in an increase of small blood vessels, selectively in the cortex. These blood vessels were dysfunctional, with a leaky blood-brain barrier (BBB), thin arteries, and accelerated hyperplasia in veins and capillaries, resembling the symptomatology of a moyamoya disease-like vasculopathy. Additionally, the research group found that both MMA patients and Neo1 mutant mice exhibited altered gene expression in the cerebral cortex in proteins critical for both angiogenesis [e.g., an increase in vascular endothelial growth factor A (VEGFA)], and axon guidance (e.g., netrin family proteins) and inflammation. In aggregates, these results suggest a critical role of astrocytic NEO1-loss in the development of a moyamoya disease-like vasculopathy, providing a mouse model for investigating mechanisms of a moyamoya disease-like vasculopathy (195). In 2021, Sesen et al. described urinary biomarkers that may identify MMA presence to a high degree of accuracy and sensitivity. These markers may be detected from the CNS to the urine, and may correlate with response to treatment, such as radiographic verification of revascularization. Urinary MMP-2 showed an accuracy of 91.3%, a specificity of 100%, and a sensitivity of 87.5%. The research group hypothesized that urinary proteins may constitute a new, non-invasive device which may assist in treatment, follow-up, prognosis, and diagnosis of MMA (196). In their 2021 study, Dei Cas et al. carried out a complete lipidomic analysis of MMA patient plasma through mass spectrometry and measured inflammatory and angiogenic protein levels through enzyme-linked immunosorbent assay (ELISA). ELISA showed an MMP-9 decrease in MMA patient plasma. Lipidomic analysis demonstrated a cumulative depletion of lipid asset in MMA patient plasma in comparison to healthy controls. The research group noted a decrease in peripherally circulating membrane complex glycosphingolipids, observed in MMA patient plasma, compared to healthy controls, indicative of cerebral cellular recruitment. This quantitative targeted approach showed increased free sphingoid bases, which may be related to aberrant angiogenesis. The results of the group may suggest that the lipid signature/plasma lipid profile of MMA patients may be closely associated with the condition and that a comprehensive biomarker profile may help to further elucidate the complexity of MMA pathogenesis (197). In their 2021 study, Lu et al., using plasma samples from 84 MMA patients, demonstrated that MMP-9 may serve as a biomarker for prediction of intracranial hemorrhage in MMA. The research group showed that a serum MMP-9 level >1,011 ng/ml may be an independent risk factor for hemorrhagic stroke in MMA. Also, the research group demonstrated that adult MMA patients, in comparison to pediatric MMA patients, showed an increased blood-brain barrier (BBB) permeability and MMP-9 serum level elevation. Furthermore, the group demonstrated that hemorrhagic MMA patients, in comparison to ischemic MMA patients, showed an increased BBB permeability and an elevated MMP-9 serum level (Figure 4) (198).

## Moyamoya angiopathy genetic biomarkers and single nucleotide polymorphisms

In 2011, Kamada et al. established the *RNF213* genetic variant, c.14576G>A (p.R4810K, rs112735431) (17q25.3), as the first MMA susceptibility gene (9, 51, 199). MMA single-nucleotide polymorphism (SNP) studies have predominantly focused on the relation to atherosclerosis, the endothelium, on mechanical stress on the vasculature, vascular repair genes, and angiogenesis (200). In 2012, Liu et al. (201) screened for the *RNF213* p.R4810K polymorphism in the East and in Southeast Asian populations and stated that the prevalence proportion may differ depending on the country (9). Moreover, the research group hypothesized that additional factors, including immune response and inflammation, may be associated with MMA onset (9, 201). The frequency of the p.R4810K genetic variant has been demonstrated to be significantly increased in MMS compared to controls (9, 202, 203), suggesting an involvement of the *RNF213* p.R4810K genetic variant in MMS. In contrast, in 2015, Miyawaki et al., based on their results from a small sample size, stated that the *RNF213* c.14576G>A genetic variant may not be related to MMS (9, 204). Pro-inflammatory cytokines such as IFN-γ, IFN-β, and TNF-α may synergistically activate *RNF213* transcription both *in vivo* and *in vitro* (9, 124, 205). Pro-inflammatory cytokines may decrease angiogenic activity through *RNF213* induction (Figure 4) (9).

Various linkage analyses have demonstrated the involvement of inflammatory genes in MMA. Ikeda et al. demonstrated associations with vascular wall homeostasis as well as with loci 17q25, 12p12, 10q23.31, 8q23, 6q25, and 3p24.2–p26 (5, 200, 206). The chromosomal site 3p may be a major gene locus of genes associated with various signaling pathways, particularly the *Interleukin 5 Receptor Subunit Alpha* (*IL5RA*) (3p26.2), *Transforming Growth Factor Beta Receptor 2* (*TGFBR2*) (3p24.1), *Thyroid Hormone Receptor Beta* (*THRB*) (3p24.2), *Retinoic Acid Receptor Beta* (*RARB*) (3p24.2), and *Peroxisome Proliferator Activated Receptor Gamma* (*PPARG*) (3p25.2). These genes may be related to signaling pathways that are associated with inflammation as well as angiogenesis (5, 9). Changes in protein folding and gene transcription may be associated with aberrant expression of ICAM-1, VCAM-1 and E-selectin, induced through pro-inflammatory cytokines TNF-α and IL-1β, by means of NF-κB activation (5, 207). A variety of genes involved in MMA may be associated with inflammation. Whether these genes are causative factors of MMA or a result of MMA pathogenesis, remains to be elucidated (4, 5, 119, 205, 208–210).

In 2009, Shimojima and Yamamoto suggested that *Actin Alpha 2, Smooth Muscle* (*ACTA2*) (10q23.31) may not be a major MMA responsibility gene, especially in Japanese MMA patients. The researchers stated that, with no evidence of a co-existence of MMA and thoracic aortic aneurysms and dissections (TAAD), MMA may be an isolated disorder (211). MMA patients typically present with transient ischemic attacks (TIA) during the juvenile stage of MMA, suggesting that TAAD associated with *ACTA2* mutations may not be characteristic for MMA (211). In their 2010 case-control study in 208 MMA patients and 224 healthy controls, Li et al. demonstrated that the MMP-3 5A/6A functional polymorphism in the MMP-3 promoter may be related to both familial MMA and MMA in Chinese Hans (212). In 2010 Roder et al. stated that their study results may indicate potential genetic factors associated with MMA pathogenesis. The research group indicated that *Platelet Derived Growth Factor Receptor Beta* (*PDGFRB*) (5q32) and *Transforming Growth Factor Beta 1* (*TGFB1*) (19q13.2) may be related to vascular growth and transformation mechanisms which may be associated with MMA pathogenesis (213). In 2011, Roder et al. demonstrated a new mutation (R179H, heterozygous) in exon 6 of *ACTA2* in one central European MMA patient. The group was neither able to detect other previously described mutations nor did they establish any significant *ACTA2* sequence variations (214). In 2012, Liu et al. stated that no novel genetic variants were identified in their study of the first *TGFB1* (19q13.2) exon in European MMA patients. Moreover the research group hypothesized that, due to a negative association of rs1800471 and rs1800470 in Japanese MMA patients, an association of the first *TGFB1* exon with MMA pathogenesis may be doubtful (215). In their 2013 letter to the editor, Hu et al. referred to their replication study results in 55 Han Chinese MMA patients. The research group stated that no *ACTA2* mutation was detected through genomic sequencing, confirming that *ACTA2* may not have an important function in MMA pathogenesis (216). In 2013, Wang et al. demonstrated that *RNF213* rs148731719 and rs112735431 may have a significant influence on MMA pathogenesis. In comparison to these results, the influences of *PDGFRB* (5q32), *MMP-3* (11q22.2), and *TIMP-2* (17q25.3) on MMA may be unremarkable in the Chinese Han population. No significant interaction among these five polymorphisms may be evident in MMA pathogenesis (217). In 2014, Cecci et al. confirmed that *RNF213* alterations may predispose patients of various ethnicities to MMA, and that the p.R4810K genetic variant may predispose individuals of Asian descent in the United States to MMA (218). In their 2014 study, Han et al. stated that familial MMA patients may constitute an increased percentage among MMA patients than estimated before. Despite the absence of distinct symptoms, family members of MMA patients may also be affected by MMA (219). Based on their 2015 study results, Kobayashi et al. hypothesized that *RNF213* R4810K carriers may have a decreased angiogenic capacity, suggesting an increased susceptibility to cerebral hypoxia of these carriers. The research group demonstrated that inflammation, interferons, may function as an environmental factor. The group also stated that decreased angiogenesis may be causally related to the *RNF213* AAA+ function (205). In their 2016 study, Kim et al. suggested that the c.14429G>A (p.R4810K) allele of *RNF213* may be related to Korean MMA patients. The homozygous c.14429G>A (p.R4810K) genetic variant may be associated with early-onset MMA, a poor prognosis, and severe symptomatology. The c.14429G>A (p.R4810K) homozygous genetic variant may provide a biomarker for early-onset MMA or unstable MMA with cerebral infarction in Korean MMA patients, requiring timely diagnosis and potentially revascularization surgery (220). In 2016, Shoemaker et al. performed a whole exome sequencing study in 125 MMA patients and 125 matched controls, establishing a non-*RNF213* founder mutation, an Asian, and a Caucasian subpopulation. Collapsing variant methodology ranked *OBSCN* (1q42.13), a gene associated with myofibrillogenesis, as most enriched in the non-*RNF213* founder mutation and among Caucasian cases. The most enriched variant in the non-*RNF213* founder mutation and among Caucasian cases was *ZXDC* (p.P562L) (3q21.3), associated with activation of MHC Class II. These results of the research group further support the East Asian origin of the *RNF213* (p.R4810K) variant and more exhaustively describe the genetic landscape of multiethnic MMA, detailing new, alternative genes and candidate variants which may be significant in MMA diagnosis, etiology, and the development of MMA models (209). In their 2016 meta-analysis, Sun et al. stated that *RNF213* rs112735431 may be related to an increased risk of MMA in the Japanese population, whereas combined screening with rs112735431 and rs138130613 may advance the detection rate for MMA in the Chinese population (221). *RNF213* is the major susceptibility gene of MMA patients in the Chinese population. The spectrum of rare genetic variants identified in Chinese MMA patients is diverse. Compared to MMA patients without rare *RNF213* genetic variants, p.R4810K heterozygous MMA patients were younger at diagnosis, had more familial cases, ischemia, and posterior cerebral artery involvement (222). In 2017, Guey et al. stated that *Cbl Proto-Oncogene* (*CBL*) (11q23.3) screening may be advocated in early-onset MMA, even in the absence of evident signs of a RASopathy. Identification of a pathogenic *CBL* mutation may raise questions concerning the hematological follow-up to be recommended to these patients (223). In 2017, Jang et al. suggested that, in their study cohort of 264 adult Korean MMA patients, *RNF213* p.Arg4810Lys may be the only genetic variant strongly related to MMA (224). In their 2017 meta-analysis, Liao et al. described the critical roles of *RNF213* p.R4810K in MMA, especially in familial MMA and intracranial major artery stenosis/occlusion (ICASO) in the Japanese, Korean, and Chinese population. Except for *RNF213* p.R4810K, MMA appears to be more complex in China. In addition to a distinct genetic background, other environmental or genetic factors may contribute to MMA (225). In 2017, Park et al. demonstrated that the *RNF213* rs112735431 polymorphism may be related to both non-MMA ICAD and MMA in the Korean population. Furthermore, the group hypothesized that the *RNF213* rs112735431 polymorphism may be associated with hypertension in MMA patients (226). In 2017, Park et al. confirmed that the *RNF213* 4950G>A and 4810G>A genetic variants may be related to both hemorrhagic and ischemic pediatric and adult MMA in the Korean population (227). In 2018, Duan et al. performed a two-stage genome-wide association study (GWAS) in 1,492 MMA patients and 5,084 controls, confirming an earlier demonstrated MMA risk locus on 17q25, and identifying 10 new MMA risk loci of genome-wide significance. The *RNF213* (17q25.3) rs9916351 single-nucleotide polymorphism (SNP) was demonstrated to have a more severe genetic impact on early-onset compared to late-onset MMA. An additional SNP related to MMA, *HDAC9* (7p21.1) rs2107595, had been associated with large vessel disease. Two new SNPs, *MTHFR* (1p36.22) rs9651118 and *TCN2* (22q12.2) rs117353193, were shown to be related to increased serum homocysteine levels in MMA patients. With a false discovery rate of <0.05, tissue enrichment analysis demonstrated genes of related loci to be exceedingly expressed in the immune system (208). In 2019, Tashiro et al. showed that, in contrast to MMA patients, the prevalence of the *RNF213* c.14576G>A polymorphism was significantly decreased in patients with an intracranial vertebral artery dissection. The *RNF213* gene polymorphism may preferentially be associated with cerebrovascular lesions in the anterior circulation, which originates from the primitive ICAs (228). In 2019, Peng et al. demonstrated that dysregulated genes in the peripheral blood of MMA patients may be associated with immune and inflammatory responses, and with ECM organization. In comparison to other vascular disorders, this gene dysregulation pattern may be specific for MMA. Moreover, resting natural killer cells, naive CD4 cells, and naive B cells were aberrantly disrupted in the peripheral blood of MMA patients (229). In their 2019 study, Pinard et al. analyzed exome sequencing results from 39 trios. With 12 altered genes predisposing to MMA, the research group demonstrated four *de novo* genetic variants in three genes, *SET Domain Containing 5* (*SETD5*) (3p25.3), *CCR4-NOT Transcription Complex Subunit 3* (*CNOT3*) (19q13.42), and *Chromodomain Helicase DNA Binding Protein 4* (*CHD4*) (12p13.31), which were previously regarded as unrelated to MMA. The aforementioned genes encode proteins involved in chromatin remodeling, and implicate disrupted chromatin remodeling as a molecular pathway predisposing to early-onset, large artery occlusive cerebrovascular disease. Moreover, these results may widen the spectrum of phenotypic pleiotropy because of alterations of *SETD5, CNOT3*, and *CHD4* extending beyond developmental disorders to late-onset cerebrovascular diseases, emphasizing the requirement to evaluate symptomatology up until adulthood for genes related to developmental disorders (230). In 2019, Shen et al. demonstrated two SNPs, related to CD40, to be associated with MMA (CC rs4813003 and TT rs1535045), which had been reported to be associated with Kawasaki disease. The research group proposed a correlation between an autoimmune disorder and MMA, hypothesizing that this genetic constitution may result in vascular wall pathogenesis (231). In 2020, Jee et al. performed a prospective computed tomography (CT) angiography study in 63 young adult MMA patients, suggesting that these patients may show a concomitant extracranial arteriopathy in distinct sites such as internal iliac, renal, celiac, superior mesenteric, and coronary artery stenosis. Also, the research group stated that MMA patients with an associated extracranial arteriopathy had an increased probability of occurrence of PCA involvement and diabetes mellitus. Furthermore, the group suggested that MMA patients carrying *RNF213* variants, particularly the homozygous *RNF213* p.Arg4810Lys variant, may benefit from screening for systemic arteriopathy (232). In 2020, Key et al. demonstrated that genetic ablation of several mitochondrial matrix factors, including the peptidase and AAA+ ATPase *Lonp1*, the transcription factor *Tfam*, and also the peptidase *ClpP*, may strongly induce *RNF213* transcript expression in several organs, along with other constituents of the innate immune system. Based on their results, the research group hypothesizes that mysterin takes effect if infections or mitochondrial dysfunction have induced RNA-dependent inflammation. Therefore, MMA may resemble vasculopathies which comprise altered nucleotide processing, including systemic lupus erythematosus or Aicardi-Goutières syndrome (146). In their 2020 study in 1024 consecutive Korean individuals without MMA, using multivariate logistic regression analysis, Kim et al. examined associations between posterior and anterior intracranial major artery stenosis/occlusion (ICASO), the main cause of ischemic stroke, and *RNF213* genetic variants. The research group demonstrated that the genotype frequency of *RNF213* 4863G > A may differ significantly according to the presence of posterior ICASO. The GA genotype of *RNF213* 4950G > A and GA genotype of *RNF213* 4810G > A may be more frequent in individuals with anterior ICASO (233). In their 2020 case-control study including 1,385 Chinese MMA patients and 2,903 healthy controls, Wang et al. stated that the *RNF213* p.R4810K genetic variant may be associated with an increased susceptibility to MMA in the Chinese population and may be related to an increased severity of PCA involvement and early-onset MMA (234). In their 2020 meta-analysis, Wang et al. included 4,711 MMA cases and 8,704 controls of 24 studies, evaluating seven polymorphisms in six genes, demonstrating that *RNF213* rs148731719 and rs112735431 may be positively, and that *MMP-3* rs3025058, *MMP-2* rs243865, and *TIMP-2* rs8179090 may be inversely related to MMA. Furthermore, the research group identified genetic variants involved in various pathophysiologic mechanisms, including vascular SMC and vascular endothelial dynamics (235). In 2021, Mineharu and Miyamoto stated that *RNF213* may occupy a decisive role in inflammation, cell cycle control, oxygen consumption, and lipid metabolism, and may contribute to vascular cell maintenance. GUCY1A3 may be a regulator of VSMC contraction and platelet function through the NO-sGC-cGMP signaling pathway. Mutations in *Guanylate Cyclase 1 Soluble Subunit Alpha 1* (*GUCY1A1, GUCY1A3*) (4q32.1) and *RNF213* may cause both MMA, and non-moyamoya intracranial arterial disease, pulmonary arterial hypertension, and coronary artery disease. They have significant interaction with CAV1 and NFAT1, both of which may have diverse molecular functions involving cell cycle control and immune regulation (119). In their 2021 genetic association study in 24 non-East Asian sporadic MMA patients, 2 singletons and 22 trios, constituting the discovery cohort, and 84 probands, 55 singletons and 29 trios, constituting the validation cohort, Kundishora et al. stated that their results may provide the largest data gathering in non-East Asians with sporadic MMA harboring pathogenic variants in the identical gene until now, suggesting that *Diaphanous Related Formin 1* (*DIAPH1*) (5q31.3) may be a MMA candidate gene, which may impair vascular cell actin remodeling, and which may influence future treatment strategies and clinical diagnostics (236). In their 2021 study, Sarkar and Thirumurugan demonstrated RNF213 dynamicity and a potential mechanism causing MMA. The research group hypothesized that mutant RNF213 may lead to insulin resistance independent of TNFα (237). Also, the research group stated that insulin resistance may lead to pericyte death and that its absence may cause microaneurysms, an established MMA disease phenotype (237, 238). RNF213 located in the nuclear region may be associated with immune response, obesity, defense response, stress response, DNA repair, cancer, and ubiquitin-binding (237). In 2021, Wan et al. carried out an association analysis of the major histocompatibility complex region in 755 MMA patients and 2,031 controls by means of an HLA imputation method, stating that the genetic polymorphism of HLA-B and HLA-DQA2 may be a genetically predisposing factor for MMA in the Chinese Han population, providing potential evidence for additional HLA-related studies of MMA patients in Chinese Hans, and suggesting that MMA may be an immune-mediated disorder (Figure 4) (239). In their 2021 study, Zhao et al. demonstrated that in MMA patients, genes associated with vascular remodeling, such as *Wnt Family Member 5A* (*WNT5A*) (3p14.3) and its associated regulators, may be disrupted and aberrant. The research group indicated that their results may assist in the development of potential future therapeutic targets which may promote MMA angiogenesis (240). According to their 2022 study results, Jin and Duan, using bioinformatics analysis, demonstrated that aberrant expression of hub genes and the characteristics of immune cell infiltration into the cerebrovascular tissue of MMA patients may provide a novel insight into MMA progression. Jin and Duan established nine hub genes associated with neutrophil regulation, of which *Unc-13 Homolog D* (*UNC13D*) (17q25.3 or 17q25.1) may be a promising MMA biomarker candidate to ascertain the characteristics of neutrophil infiltration in MMA (241). Referring to their 2022 *in vitro* study, using the CRISPR-Cas9 genome editing technology, Roy et al. indicated that RNF213 may be associated with the regulation of cerebral endothelium integrity, whose disruption may be a pathophysiological mechanism associated with MMA. Also, this study of the research group may emphasize the significance of BBB integrity in MMA pathogenesis and additional RNF213-related diseases (242). Regarding their 2022 transcriptomic study results, Xu et al. highlighted that mitochondrial function and extracellular matrix (ECM) organization may be central molecular mechanisms associated with MMA, and have ascertained a sex difference in gene expression in intracranial arteries. The research group indicated that sex-specific DEGs, including *Nuclear Receptor Subfamily 4 Group A Member 1* (*NR4A1*) (12q13.13), *Superoxide dismutase 3* (*SOD3*) (4p15.2), and *Aquaporin-4* (*AQP4*) (18q11.2), may contribute to the sex difference in MMA (243). In their 2022 study, Zhang et al. suggested that *RNF213* loss of function may reorganize the vascular transcriptome and spliceosome, which may lead to disrupted angiogenesis and an aggravated vascular inflammatory response (244). The research group indicated that *RNF213* gene knockdown may sensitize endothelial cells to inflammation, leading to aberrant angiogenesis (244). The group ascertained significant associations between *RNF213* genetic variants and immune and inflammatory MMA inducers, as well as regarding the mechanism of action of the MMA epitranscriptome (244).

## Moyamoya angiopathy non-coding ribonucleic acids

High-throughput sequencing has established a large quantity of distinct ribonucleic acids (RNAs) created from non-coding DNA (245, 246). Similar to protein-coding RNAs, non-coding RNAs appear to be linear molecules with 3′ and 5′ termini, which constitute defined end and start points of the RNA polymerase on the DNA template (245). Non-coding RNAs vary in length (245).

Long non-coding RNAs (lncRNAs) exceed 200 nucleotides, lack protein-coding capacity, and are associated with post-transcriptional processing, transcriptional control, and chromatin remodeling (9, 247, 248). Regulation of lncRNAs may be associated with inflammation (9, 249, 250). Moreover, lncRNAs may be related to MMA pathophysiology by means of an inflammatory signaling cascade comprising the MAPK signaling pathway (9, 10, 251). In their 2017 study, Wang et al. demonstrated that an integrated analysis of lncRNA-mRNA coexpression networks may be associated with the MAPK signaling pathway, the Toll-like receptor signaling pathway, cytokine-cytokine receptor interaction, and inflammation. The research group indicated that differentially expressed genes may help to ascertain crucial components in MMA pathophysiology (251). In their 2020 study, Gu et al. carried out a bioinformatics analysis of candidate RNAs to identify a series of aberrant 2294 mRNAs, 3649 lncRNAs, and 94 miRNAs, differentially expressed between samples of MMA patients and controls. The research group established a synergistic ceRNA lncRNA-miRNA-mRNA regulatory network. Key mRNAs (CXCL16, CEBPB, FOSL2, and STAT5B) and essential regulatory miRNAs (miR-423-5p and miR-107) related to the ceRNA network were identified. Gene Ontology (GO) and Kyoto Encyclopedia of Genes and Genomes pathway (KEGG) enrichment analyses employed with the DAVID database indicated that differentially expressed mRNAs (DEmRNAs) related to the ceRNA network may be associated with inflammation and immune system regulation in MMA. These research results may further elucidate the molecular MMA pathogenesis, and may contribute to constitute future treatment strategies (252). In 2020, Han et al. showed that metabolic adjustments by dysregulated lncRNAs in peripheral neutrophils may in part account for complete compensation of asymptomatic MMA patients (Figure 4) (253). In their 2021 study, Zhao et al. using KEGG and GO analysis, showed that lncRNAs and mRNAs may be differently expressed in the superficial temporal artery (STA) vascular wall in MMA patients. The research group proposed a list of altered lncRNAs and mRNAs associated with vascular remodeling, which may be possible targets of future exploration of MMA medication (240). In their 2022 study in 21 MMA patients and 11 controls, Mamiya et al. demonstrated that the lncRNA expression profile in MMA MCA microsamples differed from controls. The research group ascertained 308 differentially expressed lncRNAs (fold change > 2, *q* < 0.05), including 2 downregulated and 306 upregulated lncRNAs in the MCA of MMA patients. Gene Ontology (GO) analyses of potential protein-coding genes, the transcription of which may be regulated in cis through ascertained differentially expressed lncRNAs, indicated an association with branching related to blood vessel morphogenesis, positive regulation of cytokine production, the T-cell receptor signaling pathway, and antibacterial humoral response (254).

MicroRNAs (miRNAs) are endogenous, short non-coding ~23 nucleotide RNAs, which may regulate gene expression through pairing to the mRNAs of protein-coding genes to control their posttranscriptional repression or cleavage (9, 245, 255, 256). miRNAs may be of vital significance regarding the control of cell aging, differentiation, survival, and proliferation (9, 255). Additionally, miRNAs may be related to angiogenesis, neurogenesis, and inflammation (9, 257). miRNAs may regulate TLR signaling through reduction of inflammation, enhanced tissue repair, and regaining of homeostasis following tissue injury and infection (9, 258). MiR-126, miR-155, and miR-21 may be associated with inflammation and vascular disorders. To do research into the network involving miRNAs and their targets leading to a coordinated gene expression pattern may lead to results which may help establish new treatment strategies to approach both aberrant vascular remodeling and to induce neovascularization after ischemia (259). Increased expression of miRNA Let-7c and miRNA-196a2 may be used as MMA biomarkers (9, 260, 261). In their 2019 study, Lee et al. analyzed the impact of *RNF213* mutations and MMA on the profiles of cell-free miRNA and protein in patient plasma samples. Levels of selected MMA-affected miRNAs in EV-depleted plasma, extracellular vesicles (EVs), and whole plasma have been confirmed through real-time quantitative polymerase chain reaction (qPCR). The research group showed that EV-encapsulated miRNA may be utilized as non-invasive biomarkers to evaluate MMA progression (262). The changes of proteins and miRNAs ascertained may be related to signaling processes such as immune activation and angiogenesis which may further elucidate MMA pathogenesis (262). Ischemic conditioning may be used to decrease the stroke risk in asymptomatic intracranial atherosclerotic arterial stenosis (263, 264). Ischemic preconditioning involves inducing moderate ischemia to exert protective functions against following severe ischemic events. Epigenetics may be associated with the outcome and the pathophysiology of stroke. Recent research has demonstrated miRNA expression following ischemic preconditioning; miRNA profiling 3 h following ischemic preconditioning demonstrated upregulation of miRNA families miR-182 and miR-200 that have been associated with neuroprotective effects of the HIF-1 and prolyl hydroxylase 2 signaling pathways (264–266). Furthermore, ischemic preconditioning has been shown to promote anti-inflammatory mechanisms by modifying the expression of cytokines during ischemic insults, suggesting a critical role of the vasculature and endothelial cells during ischemic conditioning stimuli (264, 267). Also, ischemic post-conditioning may represent a promising neuroprotective strategy in ischemic insults by means of anti-inflammatory, anti-apoptotic, and CBF-based mechanisms (264, 268, 269). In 2014, Dai et al. using real-time PCR, identified a serum miRNA signature in MMA. The research group demonstrated in an independent MMA cohort that serum miR-125a-3p was significantly decreased, whereas serum miR-126, miR-130a, and miR-106b were significantly increased. Gene Ontology (GO) analysis demonstrated that differentially expressed serum miRNAs may be enriched in signal transduction, transcription, and metabolic processes. Pathway analysis demonstrated that the most enriched pathway may be the mTOR signaling pathway. Also, the research group demonstrated that 13 and 16 aberrant serum miRNAs coordinately inhibited BRCC3 and RNF213 protein expression at the posttranscriptional level, associated with MMA pathogenesis and aberrant angiogenesis (270). In 2015, Zhao et al. showed that increased serum miRNA let-7c expression in MMA patients may be associated with MMA pathogenesis through its influence on *RNF213*, suggesting that let-7c may be a potential biomarker of MMA (Figure 4) (260). In their 2018 MMA discordant monozygotic twin-based study, Uchino et al. confirmed a new circulating microRNA signature in MMA as a feasible diagnostic marker, which may be marginally confounded through genetic heterogeneity (271). The research group stated that this novel circulating microRNA signature may contribute to future functional microRNA analyses to ascertain novel therapeutic and diagnostic MMA targets (271).

In 2017, Zhao et al. demonstrated that various circRNAs may be involved in MMA pathogenesis, and may be associated with modulation of the MAPK signaling pathway. Besides providing a set of potential diagnostic biomarkers for MMA, the results of the research group suggest that therapeutic strategies targeting the MAPK signaling pathway or these circRNAs may be effective MMA treatment strategies (272). Recent research may provide evidence that regulatory RNAs including miRNAs or lncRNAs may be associated with MMA pathogenesis. In comparison with other kinds of miRNA sponges, circRNAs have higher expression levels and an increased amount of binding sites and, compared to linear RNAs, are viewed as more efficient regarding gene expression regulation and sequestering miRNAs (245, 273). CircRNAs have been associated with various disorders that involve various neurological disease, and are correlated with miRNA expression (273, 274). In 2017, Zhao et al. demonstrated that 146 circRNAs may be expressed in MMA patients, and these circRNAs may contribute to MMA pathogenesis (272, 273). Of these 146 circRNAs, 29 circRNAs were upregulated, and 117 circRNAs were downregulated (272, 273). Hsa_circRNA_067130, hsa_circRNA_067209, and hsa_circRNA_062557 were upregulated, while hsa_circRNA_089763, hsa_circRNA_089761, and hsa_circRNA_100914 were downregulated with highest fold variations, providing sufficient evidence to state that these circRNAs may be potential MMA biomarkers (272, 273). In their 2019 pilot study of neutrophil samples from asymptomatic MMA patients and an aberrant circRNA profile obtained through high-throughput microarray analysis, Ma et al. demonstrated a critical function of circRNAs and neutrophils in the differentiation of asymptomatic MMA patients compared to healthy controls, suggesting a relation of angiogenic and anti-inflammatory markers to asymptomatic MMA (264, 275). The research group carried out Gene Ontology (GO) and Kyoto Encyclopedia of Genes and Genomes pathway enrichment (KEGG) analyses to both predict functioning and for the annotation of differentially expressed circRNAs, stating that differentially expressed circRNAs may be associated with metabolism, angiogenesis and immune response in asymptomatic MMA. Also, the research group suggested an association of the HIF-1α signaling pathway with increased VEGF and angiogenesis in MMA pathogenesis (264, 275). Moreover, Ma et al. suggested that anti-inflammatory mechanisms and neutrophils may be associated with MMA progression (264, 275). Research results may indicate a HIF-1/VEGF mechanism associated with angiogenesis (264, 267). In their 2021 study, Li et al. conducted neutrophilic tsRNA profiling in asymptomatic MMA patients and healthy controls (276). Pathophysiological mechanisms, including immune response, angiogenesis, axon guidance, and metabolism adjustment, were highlighted through differentially expressed (DE)-tsRNAs and DE-mRNA in asymptomatic MMA patients, which may support the potential receptivity of asymptomatic MMA to medical therapeutics, such as immune-modifying drugs (Figure 4) (276).

## Pediatric and adult moyamoya angiopathy

The distinct pathophysiology of pediatric MMA compared to adult MMA is evident both in angiographic findings and based on the symptomatology (Figure 5). In 2009, Czabanka et al. demonstrated that surgical revascularization by encephalomyosynangiosis (EMS) combined with extracranial-intracranial bypass (STA-MCA bypass) may lead to favorable clinical and angiographic results in both pediatric and adult MMA patients. Especially in pediatric MMA patients, EMS may constitute an appropriate alternative to STA-MCA bypass surgery in the European population (277). In their 2012 study, Bao et al. stated that the symptomatology of adult MMA patients in the Chinese population may be different from those in other Asian countries. EDAS surgery in adult MMA patients may carry a low risk, is effective at preventing future ischemic events, and improves the quality of life. The research group indicated that, despite the lack of prospective randomized trials to determine the efficacy of bypass surgery in MMA patients, the available data may support surgical treatment (278). In 2012, Kim et al. demonstrated that adult MMA is not a syndrome, rather a readily distinguishable disease entity including significant progression of unilateral MMA to bilateral MMA, stating that treatment strategies and diagnostics in adult MMA should be different from those in pediatric MMA (279). In 2013, Hishikawa et al. assessed the angiographic correlation between the posterior circulation and the anterior circulation in adult MMA patients and pediatric MMA patients, and evaluated the presence of steno-occlusive PCA lesions and the extent of steno-occlusive ICA lesions on angiography. The research group stated that less advanced ICA lesions may significantly complicate posterior circulation involvement in pediatric MMA patients (280). In 2014, Lee et al. stated that during follow-up of pediatric MMA patients, clinicians should be aware of potentially delayed PCA involvement and progressive PCA stenosis, if these patients report transient visual symptoms or headache, stating that indirect revascularization surgery may be effective in these patients (281). In their 2015 study, Acker at al. demonstrated that the percentage of hemorrhagic MMA in pediatric patients was slightly increased. In comparison to adult MMA patients, angiographic analysis showed that stenosis and/or occlusion within the posterior circulation may be increased in pediatric MMA patients (282). In their 2015 long-term survey, Bao et al. showed that the majority of surgically treated pediatric MMA patients maintained a favorable outcome. The research group suggested that both timely diagnosis and active intervention prior to establishment of irreversible hemodynamic change may be essential to obtain a favorable clinical outcome (283). In their 2015 article, Piao et al. state that, in comparison to adult MMA, prognosis and treatment of pediatric MMA may be of considerably larger clinical significance (284). Also, the group argued that, through adequate treatment, favorable results may be obtained, referring to the fact that a standard treatment plan for pediatric MMA is currently not implemented in clinical practice (4, 284). In their 2016 study, Liu et al. stated that EDAS surgery may effectively increase cerebral blood flow and establish a favorable outcome in pediatric MMA patients, which may lead to a decreased incidence of recurrent hemorrhage and to disappearance of intracranial aneurysms (285). In their 2016 histopathological study of the distal MCA in pediatric MMA and adult MMA, Takagi et al. demonstrated that MCA medial thinning occurred in both pediatric and adult MMA patients. Yet, MCA intimal thickening was demonstrated to be more prominent in adult MMA patients. Additional study of MMA MCA specimens may be warranted to further clarify MMA pathophysiology (286). In 2017, Mejia-Munne et al. stated that juvenile-onset autoimmune disease and atherosclerosis were found to be associated with both adult and pediatric MMA. Adult-onset autoimmune disease was associated with pediatric MMA but not with adult MMA. The research group suggested that both adult and pediatric MMA may be associated with inflammation, hypothesizing that inflammation may be associated with MMA pathogenesis (287). In 2017, Uchino et al. stated that failure to notice non-focal physical symptoms, suggestive of orthostatic intolerance, including headache, motion sickness, difficulty getting out of bed, fatigue, and vertigo/dizziness, may significantly impair the quality of life in pediatric MMA patients up to 5 years after revascularization surgery, resulting in 57% of patients being unable to attend school. These symptoms, inversely associated with the number of years after surgery, may serve as independent clinical markers to monitor disease outcome (288). In their 2018 retrospective follow-up study, Bao et al. stated that EDAS surgery may be effective in a Chinese cohort of adult MMA patients. EDAS resulted in prevention of recurrent stroke and adequate long-term improvement of symptomatology. Hypertension may be a risk factor for ischemic stroke during follow-up (289). In their 2018 study, Elbers et al. suggested that lenticulostriate collaterals in children with unilateral intracranial arteriopathy may be a useful neuroimaging biomarker that may help stratify patients with distinct clinical features and patterns of vascular evolution (290). In their 2018 long-term follow-up study, Zhang et al. stated that EDAS surgery in pediatric MMA patients may be effective and safe, may improve the quality of life, and may diminish the risk of subsequent neurological events. The risk of ischemia-related complications was increased in younger patients, and older children showed more favorable outcomes. Compensation was greater with more prominent cerebral ischemia. The long-term clinical outcome largely depended on presence and extent of pre-operative stroke (291). In 2019, Lu et al. showed that the incidence of transient neurological events (TNE) was significantly increased in adult MMA patients compared to pediatric MMA patients (292). In their 2020 retrospective study in 131 adult MMA patients and 83 pediatric MMA patients, Liu et al. stated that pediatric MMA patients may show greater patency and an increased capability to establish a favorable leptomeningeal collateral status in comparison to adult MMA patients. The research group indicated that a poor leptomeningeal collateral status may correlate with an unfavorable post-operative outcome and severe symptomatology. The leptomeningeal collateral status may be associated with differences in prognosis and symptomatology between adult MMA patients and pediatric MMA patients (Figures 1–3) (293). In their 2020 letter to the editor, Yu et al. stated that the PCA-ACA/MCA anastomosis may increase the hemodynamic burden of the posterior circulation, increasing the risk of intracerebral hemorrhage (Figures 2, 3) (7, 294), which should be considered during the clinical management of pediatric MMA patients (294). Additional compensatory collaterals, including extracranial arterial collateral circulation anastomoses from the middle meningeal, maxillary and facial arteries to the ophthalmic artery, and dural arteriolar anastomoses from the occipital artery and middle meningeal artery through the parietal foramen and mastoid foramen, may as well correlate with the clinical outcome post-operatively (Figure 1) (294, 295). Most recently, the research group observed different hemodynamic sources of the recipient parasylvian continental arteries (PSCAs) among the parietal, temporal, and frontal PSCAs in MMA hemispheres (80, 294), suggesting that the recipient vessel in STA-MCA bypass surgery may not necessarily originate from the MCA (294). Consequently, neurosurgeons may be advised to rely predominantly on digital subtraction angiography (DSA) to ascertain the hemodynamic source of recipient vessels (294). In their 2021 retrospective validation and extension study on the function of the *RNF213* p.R4810K genetic variant in 2,877 Chinese MMA patients, Wang et al. stated that carrying rates and incidence of *RNF213* p.R4810K in various regions for Chinese MMA patients were obviously different. *RNF213* p.R4810K may have various predictive effects on the phenotypes of adult MMA patients and pediatric MMA patients (296). In their 2022 study in 15 pediatric MMA patients, Wang et al. stated that EDAS surgery may prevent ischemia/ischemic stroke, and may reduce aberrant collaterals and dilation of the anterior choroidal artery, potentially reducing the incidence of recurrent intracerebral hemorrhage of the posterior communicating artery or anterior choroidal artery during adulthood of these patients (297).

## Moyamoya angiopathy, moyamoya syndrome, and inflammation

Two predominant pathways have been suggested to be associated with inflammation and initiation or progression of MMA. A pro-inflammatory cytokine pathway, leading to *RNF213* activation, as well as an anti-inflammatory cytokine pathway. The pro-inflammatory pathway is associated with increased inflammatory cytokines in inflammatory diseases which affect RNF213, leading to MMS onset. This hypothesis may be supported by the evidence of an increased frequency of the *RNF213* p.R4810K variant in MMS. Late-onset MMA may be associated with this variant (9, 298–301). Pro-inflammatory cytokines may be involved in fulminant MMA progression (9, 302). This particularly applies to MMS associated with hyperthyroidism (9, 303–306). The pro-inflammatory pathway may function as an initiator of MMA (9). The anti-inflammatory cytokine pathway involves anti-inflammatory mediators in the CSF or the blood that may affect acceleration or acute aggravation of MMS. Anti-inflammatory cytokines may be involved in autoregulation as well as vascular reactivity, leading to MMA progression (Figure 4) (9).

In their 2002 study, Soriano et al. using dual-antibody enzyme-linked immunoassays, demonstrated increased CSF levels of soluble endothelial adhesion molecules, vascular cell adhesion molecule Type 1 (VCAM-1), intercellular adhesion molecule Type 1 (ICAM-1), and E-selectin, suggesting that pediatric MMS patients may have persistent central nervous system inflammation, with marginal blood-brain barrier (BBB) impairment. The research group suggested that these soluble adhesion molecules may be of use in clinical practice as markers of this central nervous system inflammation process. Moreover, the research group stated that their results may not completely ascertain an association of these adhesion molecules with vascular pathological processes related to MMS, since cerebral ischemia as well may lead to expression of these adhesion molecules (110). In their 2011 case-control study in 114 pediatric MMA patients and 114 healthy controls, Li et al. stated that increased thyroid autoantibodies and elevated thyroid function may be related to MMA (307). In their 2013 retrospective study, Li et al. stated that MMA associated with Graves' disease may mainly be observed in adult female patients. Associated clinical symptoms may include ischemia and may be related to hyperthyroidism. MMA pathogenesis associated with Graves' disease may be related to various immunologic and genetic influencing factors. Encephaloduroarteriosynangiosis (EDAS) surgery may diminish the likelihood of stroke recurrence and may lead to an effective collateral circulation (308). In their 2015 retrospective study, Han et al. stated that the radiographic and clinical characteristics of neurofibromatosis type I (NF-1) in MMS may be similar to MMA (309). In their 2015 case report, Hyakuna et al. described a rare case of *Cbl Proto-Oncogene (CBL)* (11q23.3) mutation related to MMA, hypothesizing that MMA could be induced through congenital dysregulation of cerebral angiogenesis associated with a RAS/MAPK pathway germline mutation. Furthermore, the research group hypothesized that prolonged RAS pathway signaling may lead to cerebrovascular development disruption (310). In their 2016 retrospective study, Acker et al. stated that their European Caucasian MMS cohort may show various disparities in comparison to a European Caucasian MMA cohort as well as to Asian MMS cohorts, hypothesizing that MMS may represent an independent disorder with a distinct ethnic symptomatology. Moreover, the research group stated that it may be important to standardize inclusion criteria and definition of MMS regarding associated disorders to optimally compare MMS results (311). In her 2016 case report about an 8-year-old female patient with a *Dedicator Of Cytokinesis 8* (*DOCK8*) (9p24.3) deletion, AlKhater stated that her patient was diagnosed with MMA (3, 312). DOCK8 deficiency may be associated with MMA (312, 313), potentially due to ischemia (312). Recent reports have described an underlying autoimmune disease mechanism related to T cell dysregulation in these patients, particularly in unilateral MMA, as evident in her patient (312, 314). Knowledge of therapy management and revascularization procedures for patients like the one described in this report remain to be ascertained (312). Prescribing antiplatelet drugs to patients affected with DOCK8 deficiency should be done with caution, due to hemorrhage being an ascertained symptomatology of MMA, particularly of hemorrhagic MMA (312, 315). In 2015, Chen et al. performed a retrospective study in 68 unilateral MMA patients and 316 bilateral MMA patients, suggesting a higher overall autoimmune disease prevalence in unilateral MMA compared to bilateral MMA. The research group hypothesized that, compared to bilateral MMA, unilateral MMA may be related to autoimmune disease to a greater extent. Moyamoya vessel formation in bilateral MMA and unilateral MMA may be associated with distinct pathogenetic mechanisms (314). Regarding their 2016 imaging study in 21 angiographically proven MMA patients, 14 MMS and 7 MMA patients, Yu et al. stated that differentiating MMS from MMA may be challenging, and high-resolution magnetic resonance imaging (HR-MRI) may help provide a more detailed comprehension of MMS and MMA, which may lead to a more precise diagnosis of increased reliability (316). In 2017, Zhang et al. showed that, in comparison to other MMA patients, the *RNF213* p.R4810K genetic variant may be related to autoimmune and atherosclerotic MMS in the Chinese population at a lower prevalence (202). In 2018, Yamanaka et al. hypothesized that HIV-associated vasculopathy, a cerebrovascular disease associated with HIV-1, caused through endothelial dysfunction, due to cytokine imbalances and inflammation related to HIV-1, may contribute to intracerebral hemorrhage and collateral vessel impairment, although the pathophysiologic mechanism of vascular damage in HIV-1 remains to be fully ascertained. Thus, adequate management of HIV-1 may be essential in MMS (317). In their 2019 moyamoya multicenter study, Bonasia et al. ascertained three types of anastomoses between the anterior and posterior cerebral circulation, consisting of collaterals from the posterior choroidal arteries (20%), the posterior callosal artery (20%), in addition to a potential pio-pial anastomosis between cortical collaterals of the posterior cerebral artery (PCA) and the anterior cerebral artery (ACA) (15%), with a distinct capacity for retrograde compensation of the anterior circulation. In advanced Suzuki stages from IV to VI in particular, collaterals are frequently observed in MMA. Collaterals may develop due to their ability to compensate the leptomeningeal anastomosis, duro-pial anastomosis, and the ophthalmic-ACA anastomosis collateral systems and due to a diminished blood supply to the ACA territory. The research group suggested a 4-grade classification based on the capability of the three types of PCA-ACA anastomoses to provide retrograde supply to the ACA territory (Figures 2, 3) (7). Based on their 2019 study in 48 MMS patients and 137 MMA patients, Feghali et al. stated that MMS patients and MMA patients may present with similar angiographic phenotypes and similar symptomatology, and may have an equally favorable outcome of surgical revascularization (318). Differentiation of MMS from MMA is important. Whereas the causes of MMS may be reversed by medication, MMA may require surgical revascularization (57). In 2020, Aloui et al. performed a rare *de novo* candidate copy number variant (CNV) screening in 13 MMA trios by use of whole genome high density single-nucleotide polymorphism (SNP) array data and whole exome sequencing (WES) reads depth data. WES and SNP array data of 115 unrelated MMA patients were used to detect recurrence of rare *de novo* CNVs, suggesting that recurrence of the Xq28 candidate CNV, its familial segregation in two additional families, and its *de novo* occurrence in one MMA patient may indicate pathogenicity. Relation of the Xq28 CNV to pulmonary hypertension and use of genetic counseling may be of relevance in clinical practice. The research group has demonstrated a new Xq28 CNV gain in both MMA and a novel MMS related to pulmonary vein stenosis, pulmonary hypertension, and other distinct systemic venous anomalies. These data may be relevant for clinical care and genetic counseling (319). MMA patients may present with a significantly increased rate of persistent carotid-vertebrobasilar anastomoses compared to controls (320), and may be 26 times more likely to suffer from Down's syndrome (321, 322). Accordingly, compared to controls, Down's syndrome patients may present with significantly increased stages of MMA, and may be more than 10 times as likely to show aberrations of the Circle of Willis (323), and vertebral arteries (322, 324). Several genes on chromosome 21 may be associated with angiogenesis, including *Down Syndrome Critical Region 10* (*DSCR10*) (21q22.13), *Dual Specificity Tyrosine Phosphorylation Regulated Kinase 1A* (*DYRK1A*) (21q22.13), and *Collagen Type XVIII Alpha 1 Chain* (*COL18A1*) (21q22.3), possibly by VEGF inhibition (322, 325, 326). In 2021, Kim et al. described a Korean female pediatric patient with CHOPS syndrome accompanied by systemic vasculopathy. The patient had been diagnosed with MMA at 6 years of age and had undergone three synangiosis surgeries. The infrarenal aorta and the renal arteries were diffusely narrowed. A new *AFF4* c.758C > T (p.Pro253Leu) missense variant was ascertained through whole exome sequencing. Except for the *RNF213* c.14429G > A (p.Arg4810Lys) genetic variant, inherited from an asymptomatic mother, no additional candidate variants associated with the vascular manifestation of this patient were identified (327). In 2021, Pinard et al. suggested a new syndrome related to *RNF213* rare variants characterized by *de novo* mutations disrupting highly conserved amino acids in the RING domain as well as a discrete region distal to the RING domain delimited by amino acids 4,114–4,120, causing early-onset, severe MMA before 3 years of age and occlusion of additional arteries, e.g., the femoral artery, iliac artery, renal artery, and the abdominal aorta (328). In 2021, Sharina et al. demonstrated that decreased cGMP-forming activity of the rare *GUCY1A3* α1C517Yβ1 sGC genetic variant may be aggravated due to reduced protein stability and increased susceptibility to oxidative stress. Combination of these deficiencies may contribute to the severity of symptoms of achalasia and MMA evident in human carriers of the α1C517Yβ1 sGC variant (Figure 4) (329).

## Conclusion, treatment strategies, and future research perspectives in moyamoya angiopathy

We have reviewed the physiological and pathophysiological mechanisms of signaling pathways, cells, and genes involved in MMA and MMS and their association with aberrant angiogenesis and inflammation (Figure 4). If mediators involved in these mechanisms are associated with signaling pathway activation or if they constitute downstream mediators remains to be elucidated (5). To do research into the effects of signaling molecules involved in MMA and the part of a signaling pathway they act, may be advocated (5). Moyamoya collateral vessel formation seems to be subsequent to ICA stenosis (5). Thus, prevention of the above-mentioned stenotic process may help avoid the subsequent formation of fragile moyamoya collaterals (5). Angiogenesis in MMA may be either decreased or facilitated (9). Research results indicate that aberrant angiogenesis, decreased or facilitated, may be associated with MMA pathogenesis. These findings seem to be validated by revascularization surgery for MMA, by which increased angiogenesis and an improved formation of a collateral circulation is achieved by restoration of blood flow to the brain (9). Despite a limitation of the number of cases involved, consensual evidence of inflammation in MMA appears to be present (5). Inflammation in pediatric stroke is critically important, both due to the inflammatory signaling cascades activated through ischemia and because of inflammatory baseline pathologies causing stroke (47). Reciprocal action of such fundamental pathophysiologic mechanisms may be of substantial importance, warranting further research (47). Focal pathophysiology may be associated with proximal vessels, such as the circulus arteriosus cerebri, the MCA (M1), ACA (A1), and the distal ICA, whereas generalized pathologies may affect small arteries or peripheral vessels (47, 330). Considerable differences in inflammatory signaling cascades in the neonatal and the adult brain are evident (47). Developmental trajectories of inflammatory signaling cascades from the neonate to the adult remain to be ascertained. Therapeutic interference with such an inflammatory pathology might be feasible by means of additional studies (47). Animal models are warranted to ascertain if these findings may be involved in MMA pathogenesis (5). Regardless whether these processes may induce MMA or result from the arteriopathy, there is growing evidence of a reversible inflammatory process being present in the vascular wall which may contribute to lumen stenosis (5). Inflammation, although not a direct cause of MMA and MMS, may influence *RNF213*, and thus result in aberrant angiogenesis (9).

### Moyamoya angiopathy treatment strategies

Enhanced interaction between neurons and cells of the vasculature, increased angiogenic activity, induced curative angiogenesis, and increased formation of a collateral circulation may be Research Topics fundamental to establishing future treatment strategies (9).

In 2011, Li et al. suggested that monitoring of thyroid autoantibodies and thyroid function in MMA patients may be advocated to assist in continuing medical treatment (307). In their 2013 study of a BALB/C male mouse model of ischemic stroke, Rosell et al. researched if treatment with EPCs or their secreted factors may intensify neurogenesis and angiogenesis after persistent focal cerebral ischemia. The research group demonstrated that applying EPC-secreted factors may be an effective and safe cell-free potential future treatment strategy for stroke (158). In 2014, Han et al. suggested that routine screening may be warranted for all family members of familial MMA patients to increase the detection rate for this patient group. In MMA diagnostics, transcranial Doppler sonography may correlate well with magnetic resonance angiography (MRA). Being safe and cost-effective, transcranial Doppler sonography may be the favored screening modality (219). In their 2014 article, Wan and Duan suggested that in hemorrhagic MMA patients, quality of life and cognitive function should be assessed and integrated into evaluations of treatment strategy effectiveness. Also, the researchers indicated that revascularization surgery may be more favorable for hemorrhagic MMA patients, and that combined bypass may lead to a more favorable revascularization and AChA-PCoA extension improvement (94). In 2015, Han et al. suggested that routine vascular screening for NF-1 MMS patients may be advocated regarding early detection of MMS as well as of other cerebral arteriopathies. The research group stated that revascularization surgery may prevent progression of clinical symptoms and diminish the likelihood of subsequent stroke in NF-1 MMS patients (309). In 2015, Hyakuna et al. hypothesized that allogeneic hematopoietic stem cell transplantation may remedy MMA pathophysiology. Niemeyer et al. (331) established a relation between vasculitis and *CBL* germline mutation (310). Although the function of mutated *CBL* in MMA and vasculitis remains to be elucidated, hematopoietic stem cell transplantation may reduce the likelihood of vasculitis (310). In 2018, Duan et al. demonstrated various new MMA susceptibility genes to be associated with homocysteine metabolism. Furthermore, due to enrichment of the expression of these susceptibility genes in the immune system, the research group suggested that therapeutic interventions directed at those pathways could be efficient MMA treatment approaches (208). In 2018, Ishii et al. stated that, in case the post-operative serum level of matrix metalloproteinase (MMP)-9 and Occludin (OCLN) may be significantly elevated, systolic blood pressure should be continuously controlled to avoid post-operative intracranial hemorrhage and/or epilepsy. Particularly regarding MMP-9, the administration of minocycline may be considered (191, 332). In 2018, Wang et al. suggested that hemorrhagic MMA patients should undergo lifelong follow-up, even if their neurological status is excellent (98). In 2019, Nishihiro et al. demonstrated that High-mobility group box-1 (HMGB1) with encephalo-myo-synangiosis (EMS) in a chronic hypoperfusion model promoted cerebral angiogenesis in a VEGF-dependent manner, resulting in improvement of cerebral blood flow. This treatment may be an effective therapy for MMA patients (333). In 2020, Zhao et al. demonstrated that autogenous bone marrow stem cell mobilization combined with dexamethasone antiinflammation and anti-infection treatment after revascularization in MMA patients may accelerate recovery of nervous function and promote blood vessel formation. At the same time, this treatment approach may reduce inflammation and improve the quality of life of MMA patients (334). In 2020, Gu et al. stated that two potential small molecule drugs, indirubin and CAY-10415, were recognized as MMA candidate drugs through Connectivity Map (CMap) (252). In 2021, Mineharu and Miyamoto suggested various treatment strategies including pharmacological eNOS-sGC-cGMP pathway stimulation, inflammation control, avoidance of hypoxia, homocysteine control, and blood lipid control. Pharmacological treatment candidates of MMA may be homocysteine lowering drugs, such as vitamin B12 or folate, lipid lowering drugs, such as Proprotein convertase subtilisin/kexin type 9 (PCSK9) inhibitors and statins, and anti-inflammatory drugs, such as anti-IL-6 antibodies or COX-2 inhibitors. Soluble guanylate cyclase stimulator Riociguat, used for pulmonary arterial hypertension treatment, could be an alternative (119, 335). Yet, there is insufficient proof of both interaction and functionality of these candidate markers, which may be associated with MMA. Identification of detailed molecular networks may acquire novel therapeutic strategies (5, 119). In their 2022 review article, Zhang et al. stated that, in addition to various pre-existing MMA staging systems, which are based on medical imaging and symptomatology, a suitable MMA grading system, capable of ascertaining MMA disease progression, may be warranted (336). Referring to their 2022 study results, Wang et al. indicated that timely indirect surgery may be warranted in pediatric MMA patients. Even though the results of the research group did not directly prove efficacy in preventing recurrent intracerebral hemorrhage, the group stated that aberrant collaterals of the posterior choroidal artery had decreased post-operatively. All subjects had a favorable clinical outcome (297).

### Moyamoya angiopathy future research perspectives

Recent MMA research may concentrate on the three main sectors therapy, prognosis, and diagnosis (6, 337). MMA therapeutic innovation research has remained behind the significant achievements in the diagnostic and prognostic area of MMA research (6, 337). Prognosis of MMA has been advanced through non-invasive biomarkers and new imaging methodologies (2, 6, 64, 196, 290, 338). MMA diagnosis has profited greatly from the latest advancements in molecular genetics, with significant progress in the identification of specific genetic variants related to clinical phenotypes and radiographic presentations (6, 51, 230, 236, 339).

Genetic analysis of familial MMA may help to ascertain the pathogenesis of MMA (4). In case of identification of relevant genes, development of novel gene therapies and prevention of MMA occurrence in genetically susceptible individuals may be possible (4). Also, the Japan Adult Moyamoya Trial (4, 99, 340–345) may contribute to ascertain the advantages of combined or direct bypass surgery for the prevention of recurrent hemorrhage in MMA (4). Additional follow-up and epidemiological studies are warranted to ascertain the pathogenesis of asymptomatic MMA (4). These results will be important to refine the guidelines for surgical and medical MMA treatment, in particular for asymptomatic or hemorrhagic MMA patients (4).

In 2002, Soriano et al. suggested that additional research may be warranted to ascertain if soluble endothelial adhesion molecules may be potential therapeutic or diagnostic markers for MMS therapeutic management (110). In 2008, Jung et al. suggested that further prospective studies may be warranted to ascertain if alterations in functioning and number of circulating EPCs may serve as prognostic or diagnostic biomarkers in MMA (159). In 2009, Shimojima et al. stated that further studies are required to identify a major disease-causing gene for MMA (211). In 2010, Kim et al. suggested that additional research may be warranted to ascertain the distinct functioning of EPCs in MMA pathogenesis (153). In 2011, Ni et al. indicated that further research may be warranted to ascertain the correlation between SDF-1α and CD34+ CXCR4+ cells in MMA (152). In 2012, Bao et al. stated that randomized clinical trials may be warranted to study the efficacy of revascularization procedures (278). In 2013, Chen et al. suggested that transfer function analysis derived phase shift and rate of recovery (RoRc) may be advantageous in clinical studies researching into hemodynamic compromise, as these may provide a both non-pharmacologic and non-invasive method with reliable sensitivity in correlation with angiography (65). In 2013, Hitomi et al. demonstrated that iPSECs may serve as an *in vitro* MMA model, expressing a useful benchmark phenotype for high throughput screening, which may be applied to drug development and used to ascertain MMA causative factors (187). In 2013, Hu et al. suggested that larger studies may be warranted to ascertain the potential association of *Actin Alpha 2, Smooth Muscle (ACTA2)* (10q23.31) and/or additional genes and MMA in different populations (216). In 2014, Dai et al. suggested that research into established angiogenesis-related genes may be a path to ascertain potentially unknown angiogenesis-associated miRNAs. Furthermore, adjustment of the pathophysiologic mechanism associated with the functioning of serum miRNAs in MMA may be a possible therapeutic strategy which may warrant additional research (270). In 2014, Kang et al. stated that their research into SPCs from the peripheral blood of MMA patients may supply a new experimental cell model for future MMA research (149). In 2014, Ryoo et al. suggested that distinct radiological findings may further elucidate MMA pathogenesis and distinguish ICAD from MMA (85). In 2014, Wan and Duan stated, that hemorrhagic MMA treatment strategies may not be standardized, and that randomized, prospective, large sample clinical trials may assist to ascertain the most favorable treatment approach (94). In 2015, Baltsavias et al. suggested that a more thorough understanding of the moyamoya collateral circulation and anastomotic networks may assist in the definition of a novel MMA staging system which may impact clinical practice (69). In 2015, Ganesan et al. suggested that addressing three distinct research areas may assist in further elucidating MMA pathogenesis (337). First, precise clinical and radiological phenotyping of distinct MMA populations to encourage research into genetic and biological mechanisms (337). Second, development of new, standardized prognostic methods to instruct therapeutic decision making and stratify the risk of disease (337). Third, prospective analysis of the efficacy of MMA surgical revascularization, to contrast the risks of distinct treatment strategies, through application of standardized radiographic, neurocognitive, and clinical assessments, in order to unbiasedly assess the efficacy of various treatment strategies (337). In 2015, Karunanithi et al. suggested that their study results may warrant further research into pressure drop indicator (PDI) as a causal factor regarding post-operative complications in MMA patients (70). In 2015, Kobayashi et al. suggested that the pathology related to aberrant SMC proliferation and decreased angiogenesis, such as arterial stenosis in the Willis' circle or moyamoya vessel formation should be addressed in future studies. Furthermore, this group hypothesized, that research into the function of *RNF213* in maintenance and remodeling of the vascular system may help elucidate mechanisms of both cerebral artery stenosis in general and in MMA (205, 346). An ATP binding inhibitor specific to the Walker A motif in the first AAA+ may be an auspicious therapeutic candidate, potentially increasing CNS hypoxic tolerance in *RNF213* R4810K carriers (205). In 2016, Duan et al. stated that the specific function of the posterior communicating artery, anterior choroidal artery, and moyamoya collaterals need to be further investigated. Also, the research group suggested that additional research on cerebral microbleeds may be warranted to further elucidate the pathogenesis of pediatric hemorrhagic MMA. Furthermore, the group indicated that comprehensive, large-sample studies may be warranted to further ascertain pediatric hemorrhagic MMA (96). In 2016, Gao et al. suggested that doing research into the MAPK signaling pathway and lncRNAs, and their potential function as therapeutic targets, may be warranted (10). In 2016, Hamauchi et al. stated that additional research may be advocated to further elucidate the pathology of differentially expressed ECM receptor-related genes and splicing regulating proteins in MMA pathogenesis (166). In 2016, Liu et al. suggested that more detailed and longer angiographic and clinical follow-up study may be warranted to ascertain the pathophysiologic mechanism underlying recurrent intracranial hemorrhage in hemorrhagic MMA (97). In 2016, Mossa-Basha et al. suggested that, in case of confirmation in larger studies, the criteria for MMA and the moyamoya diagnostic algorithm may be revised according to improved diagnostic accuracy in addition to a potential limitation of invasive diagnostics (83). In 2016, Scholz et al. stated that the identification of an association between *RNF213* and NFAT1 may be another method for further research into the molecular pathogenesis of MMA (120). In 2017, Liao et al. suggest that studies identifying the ethnicity-specific factors and pathological role of *RNF213* genetic variants in MMA and intracranial major artery stenosis/occlusion (ICASO) may be warranted (225). In 2017, Mejia-Munne et al. suggested, that additional research may be warranted to ascertain the pathophysiology of MMA and inflammation (287). In 2017, Park et al. suggested that additional studies may be warranted to ascertain if the *RNF213* rs112735431 polymorphism may be related to hypertension in MMA patients and healthy controls in the Korean population. Definition of the relationship between the *RNF213* rs112735431 polymorphism and hypertension in MMA patients as well as determination of the specific biochemical function of *RNF213*, which may be involved in the pathogenesis of hypertension, may be advocated (226). In 2017, Qiao et al. suggested that studies to ascertain methods to evaluate the moyamoya collateral circulation by use of combined multimodality imaging techniques, such as perfusion imaging, structural MRA imaging and functional brain imaging, to assess cortical structural change as a consequence of revascularization surgery, may provide results which may assist in clinical decision making, including patient selection strategies for operative management of MMA patients (89). In 2017, Uchiyama stated that study of the *RNF213* genetic variant in twig-like MCA patients may clarify the twig-like MCA pathogenesis, enabling to establish a differential diagnosis of MMA (90). In 2017, Wang et al. suggested that future research may concentrate on the MAPK signaling pathway and on inflammation in MMA (251). In 2018, Ishii et al. suggested that histopathologic examination of the blood-brain barrier (BBB) and quantitative assessment of cerebral blood flow and cerebral blood volume may be warranted for validation of their hypothesis (191). In 2019, Liu et al. stated that longer follow-up studies and larger patient samples may be warranted to substantiate the value of their proposed new MMA collateral grading system (79). In 2019, Tashiro et al. suggested that the genetic background underlying intracranial vertebral artery dissection should be elucidated in future studies (228). In 2019, Corey and Luo suggested that research into the involvement of neutrophils in moyamoya progression could be an additional path for future research (264). According to their preliminary 2019 cohort study results, Funaki et al. suggested that verification of their present results in larger studies and additional research on the effect of choroidal collaterals on recurrent intracranial hemorrhage in hemorrhagic MMA may be warranted to ascertain the best possible treatment strategy for asymptomatic MMA patients and for non-hemorrhagic cerebral hemispheres in adult hemorrhagic MMA patients in the Japan Adult Moyamoya (JAM) Trial (99). In 2019, Shen et al. suggested continued future research to investigate if CD40 may function as a personalized MMA marker (231). In their 2019 article, Young et al. stated that further prospective studies may be warranted to evaluate the clinical utility of intracranial vessel wall imaging in differentiating MMS from MMA, and to predict hemorrhage and ischemia, which may help identify high-risk and low-risk patients and direct clinical management (57). In 2019, Bonasia et al. suggested that both an analysis of the three types of anastomoses between the anterior and posterior cerebral circulation by selective contrast injection into the PCA and an analysis of the ophthalmic-ACA anastomosis, the leptomeningeal anastomosis, and the duro-pial anastomosis collateral systems may be warranted to further elucidate MMA pathogenesis and to better identify patients who may benefit from bypass surgery (7). In 2020, Aloui et al. suggested that additional research may be warranted to further elucidate the mechanism associated with the Xq28 candidate copy number variant and the pathogenesis of vascular disease in patients affected with MMA and a novel MMS (319). In 2020, Han et al. suggested that platelet activation and renin secretion may help guide clinical management and may further elucidate the pathogenesis of asymptomatic MMA (253). In 2020, Kim et al. suggested that additional study of the molecular biology and functioning of *RNF213* may further ascertain the pathophysiology of cerebrovascular disease and ICASO (233). In 2020, Wang et al. stated that larger cohorts, including different ethnicities, may be warranted to further clarify associations between *TGFB1* SNPs and MMA (235). Also, with both genetic and environmental factors being associated with MMA pathogenesis (347), multivariate analysis to adjust confounders, including biochemical, clinical, and behavioral factors, should be incorporated in future studies (235). In 2021, Byworth et al. suggested that further research may be warranted to ascertain whether additional copies of the *Down Syndrome Critical Region 10* (*DSCR10*) (21q22.13), *Dual Specificity Tyrosine Phosphorylation Regulated Kinase 1A* (*DYRK1A*) (21q22.13), and *Collagen Type XVIII Alpha 1 Chain* (*COL18A1*) (21q22.3) genes may be associated with aberrant vascular development in Down's syndrome, which may predispose to MMA (322). In 2021, Han et al. suggested that methods to alleviate inflammation and to restore the BBB may be efficient MMA treatment strategies (194). In 2021, Li et al. stated that additional studies may be warranted to help clarify the pathophysiologic mechanism associated with neutrophilic tsRNAs and their associated signaling pathways in asymptomatic MMA patients, to further elucidate MMA pathogenesis (276). In 2021, Lu et al. suggested that the serum levels of BBB-related proteins and MMP-9, in addition to their comparison between MMA subgroups, should be compared to healthy controls (198). Also, the research group indicated that the pharmaceutical significance of a strengthened impact of MMP-9 on surgery and the predictive value of intracranial hemorrhage prediction should be subject to validation in future research (198). Moreover, the group stated that additional research may be warranted to further ascertain the function of MMP-9 and BBB impairment in MMA pathophysiology (198). In 2021, Mineharu et al. stated that the functions of *GUCY1A3* and *RNF213* have been intensively studied in VSMCs and vascular ECs. However, with the distinct mechanism of fibrosis and intimal thickening in MMA remaining to be elucidated, research into the function of GUCY1A3 and RNF213 in immune cells, especially in dendritic cells, neutrophils, B cells, and T cells may be warranted. Additional vascular components, including the extracellular matrix (ECM), platelets, and inflammatory cells, should be subject of future research (119). In 2021, Sarkar et al. stated that further *RNF213* knockdown studies may be warranted to confirm both the function of *RNF213* in TNFα/PTP1B mediated obesity and insulin resistance and detailed pathophysiologic mechanisms related to this signaling pathway (11). In 2021, Sarkar et al. suggested that doing research on the effect of iron-binding in MMA and on the pathophysiologic mechanism of RNF213 in cancer and obesity may be warranted (237). In 2021, Wu et al. stated that additional prospective studies may be warranted to further ascertain the association between bleeding spots and aberrant MMA collaterals (103). Relating to their 2022 study results, Jin and Duan stated that, due to the complex functions and molecular genetic mechanisms, their bioinformatics results may warrant verification experiments. Jin and Duan hypothesized that the slow progression of MMA may be associated with a distinct gene expression at each MMA stage, that the genetics of adult MMA and pediatric MMA may be different, warranting additional clarification of such potential variations (241). Referring to the results of their 2022 transcriptome-wide analysis, Xu et al. suggested that the sex difference should be considered in future MMA research (243).

Continued research into MMA pathophysiology and associated signaling pathways may identify new treatment strategies, therapeutic applications, and mechanism-tailored interventions that may halt MMA progression. Research into EPCs, endothelial cells, and pericytes may further elucidate the function of vasculogenic, angiogenic, and anti-angiogenic markers and associated signaling pathways (3, 348). Reduction of interlaboratory variations and methodological differences may facilitate cooperation between laboratories (3, 348, 349). Constant evaluation of novel prognostic and diagnostic resources obtained through research may help to effectively and safely transfer research results into practice (2). Ongoing collaborative, prospective basic laboratory, and large-scale, large cohort clinical research on pathophysiologic mechanisms, a multi-professional, multi-center, international collaboration between vascular and stroke physicians, and clinician-scientists pursuing translational research are essential to establish large biorepository, imaging, and clinical data sets, which may be required if we are to better understand the complex etiology of MMA, potentially leading to increasingly differentiated diagnoses and disease-modifying treatment strategies (2, 6, 44, 92, 337, 350).

## Author contributions

KD contributed to developing the concept of the review, developing the figures, and writing and editing the manuscript. JW oversaw the project and contributed to developing the concept of the review, developing the figures, and editing the manuscript. Both authors contributed to the article and approved the submitted version.
