# Supplementary material for: Physiological and pathophysiological mechanisms of the molecular and cellular biology of angiogenesis and inflammation in moyamoya angiopathy and related vascular diseases
Source: Front Neurol. 2023 May 16;14:661611. doi: 10.3389/fneur.2023.661611 (PMC10236939; doi:10.3389/fneur.2023.661611)
Supplement: Supplementary file 1 [file Table_1.docx]

**Supplemental Table 1.** Abbreviations and *gene symbols*

| **Abbreviation** | **Definition** | |
| --- | --- | --- |
|  |  | |
| AAA+ | AAA ATPase domain | |
| ACA | Anterior cerebral artery | |
| AChA | Anterior choroidal artery | |
| *ACTA2* | Alpha-actin-2 | |
| ACVD | Atherosclerotic cerebrovascular disease | |
| Akt | Protein kinase B | |
| ANG | Angiopoietin | |
| APA | Anterior pericallosal artery | |
| ATP | Adenosine triphosphate | |
| BA | Basilar artery | |
| BBB | Blood-brain barrier | |
| bFGF | Basic fibroblast growth factor | |
| BMCPC | Bone marrow-derived circulating progenitor cell | |
| BMP4 | Bone morphogenetic protein 4 | |
| BRCC3 | Lys-63-specific deubiquitinase BRCC36 | |
| *BRCC3* | BRCA1/BRCA2-containing complex subunit 3 | |
| Ca2^+^ | Calcium | |
| *CAV1* | Caveolin 1 | |
| CBF | Cerebral blood flow | |
| CBL | E3 ubiquitin-protein ligase CBL | |
| *CBL* | Cbl proto-oncogene | |
| CCL | C-C motif chemokine | |
| CCR | CCL receptor | |
| CD | Cluster of differentiation | |
| CEBPB | CCAAT/enhancer-binding protein beta | |
| *CEBPB* | CCAAT Enhancer Binding Protein Beta | |
| ceRNA | Competing endogenous RNA | |
| CFU | Colony forming unit | |
| *CHD4* | Chromodomain helicase DNA binding protein 4 | |
| CHOPS | Cognitive impairment and coarse facies-congenital heart defect-obesity-pulmonary involvement-short stature and skeletal dysplasias | |
| circRNA | Circular RNA | |
| ClpP | Caseinolytic peptidase P | |
| CMA | Calloso-marginal artery | |
| CMC1 | COX assembly mitochondrial protein homolog | |
| *CMC1* | C-X9-C Motif Containing 1 | |
| *CNOT3* | CCR4-NOT transcription complex subunit 3 | |
| CNS | Central nervous system | |
| CNV | Copy number variant | |
| *COL18A1* | Collagen type XVIII alpha 1 chain | |
| COX | Cyclooxygenase | |
| CRABP1/*CRABP1* | Cellular retinoic acid-binding protein 1 | |
| CSF | Cerebrospinal fluid | |
| CT | Computed tomography | |
| CTNNB1 | Catenin beta-1 | |
| *CTNNB1* | Catenin beta 1 | |
| CXCL5 | C-X-C motif chemokine ligand 5 | |
| CXCR | C-X-C chemokine receptor | |
| 3D-CISS | Three-dimensional constructive interference in steady state | |
| DEmRNA | Differentially expressed mRNA | |
| *DOCK8* | Dedicator of cytokinesis 8 | |
| DSA | Digital subtraction angiography | |
| *DSCR10* | Down Syndrome Critical Region 10 | |
| *DYRK1A* | Dual specificity tyrosine phosphorylation regulated kinase 1A | |
| EC | (Vascular) endothelial cell | |
| ECA | External carotid artery | |
| ECFC | Endothelial colony-forming cell | |
| ECM | Extracellular matrix | |
| EDAS | Encephaloduroarteriosynangiosis | |
| EEG | Electroencephalography | |
| ELAM-1 | E-selectin | |
| EPC | Endothelial progenitor cell | |
| Eph | Erythropoietin-producing human hepatocellular receptor | |
| Epo(R) | Erythropoietin (receptor) | |
| ERK | Extracellular signal-regulated kinase | |
| *ETS1* | Protein C-Ets-1 | |
| EV | Extracellular vesicle | |
| FGF | Fibroblast growth factor | |
| FOSL2 | Fos-related antigen 2 | |
| FOXO1 | Forkhead box protein O1 | |
| *FOXO1* | Forkhead box protein O1 | |
| G-CSF | Granulocyte colony stimulating factor | |
| GO | Gene Ontology | |
| GTP | Guanosin triphosphate | |
| *GUCY1A3* | Guanylate cyclase soluble subunit alpha-3 | |
| GWAS | Genome-wide association study | |
| *HDAC9* | Histone deacetylase 9 | |
| HGF/*HGF* | Hepatocyte growth factor | |
| HIF-1(α) | Hypoxia-inducible factor 1(-alpha) | |
| *HIF1A* | Hypoxia-inducible factor 1 alpha subunit | |
| HIF-1β | Hypoxia-inducible factor 1-beta | |
| HIV-1 | Human immunodeficiency virus type 1 | |
| HLA | Human leukocyte antigen | |
| HMGB1 | High-mobility group box-1 | |
| HR-MRI | High-resolution magnetic resonance imaging | |
| ICA | Internal carotid artery | |
| ICAD | Intracranial atherosclerotic disease | |
| ICAM-1/*ICAM1* | Intercellular adhesion molecule 1 | |
| ICASO | Intracranial major artery stenosis/occlusion | |
| IFN-α/-β/-γ | Interferon alpha/-beta/-gamma | |
| IGF-1 | Insulin-like growth factor 1 | |
| IL | Interleukin | |
| IL5RA | Interleukin-5 receptor alpha | |
| IPC | Ischemic preconditioning | |
| iPSC | Induced pluripotent stem cell | |
| iPSEC | iPSC-derived vascular endothelial cell | |
| JAK-STAT | Janus kinase-signal transducer and activator of transcription protein | |
| KDR | Kinase insert domain receptor | |
| KEGG | Kyoto encyclopedia of genes and genomes pathway | |
| *LCN2* (*NGAL*) | Lipocalin 2 | |
| Lef-1 | Lymphoid enhancing factor | |
| lncRNA | Long noncoding RNA | |
| *LONP1* | Lon Peptidase 1, Mitochondrial | |
| *MAP2K1* | MAPK/ERK Kinase 1 | |
| MAPK | Mitogen-activated protein kinase | |
| MBFV | Mean blood flow velocity | |
| MCA | Middle cerebral artery | |
| MCP-1 | Monocyte chemoattractant protein-1 | |
| MHC | Major histocompatibility complex | |
| miRNA | MicroRNA | |
| MMA | Moyamoya angiopathy |  |
| MMP | Matrix metalloproteinase |  |
| *MMP2* | Matrix Metallopeptidase 2 |  |
| *MMP3* | Matrix Metallopeptidase 3 |  |
| MMS | Moyamoya syndrome |  |
| MPChoA | Medial posterior choroidal artery |  |
| MR(A) | Magnetic resonance (angiography) |  |
| MRI | Magnetic resonance imaging |  |
| mRNA | Messenger RNA |  |
| *MTHFR* | Methylenetetrahydrofolate reductase |  |
| mTI-ASL | Multiple inversion time arterial spin labeling |  |
| mTOR | Mammalian target of rapamycin |  |
| *NEO1* | Neogenin 1 |  |
| NF-1 | Neurofibromatosis type I |  |
| NFAT | Nuclear factor of activated T-cells |  |
| NF-κB | Nuclear factor kappa-light-chain-enhancer of activated B cells |  |
| NGAL | Neutrophil gelatinase-associated lipocalin |  |
| *NLRP3* | NLR Family Pyrin Domain Containing 3 |  |
| NO | Nitric oxide |  |
| NO-sGC-cGMP | Nitric oxide-soluble guanylyl cyclase-cyclic guanosine monophosphate |  |
| *NOTCH2* | Notch homolog 2 |  |
| *NRAS* | Neuroblastoma RAS viral oncogene homolog |  |
| *OBSCN* | Obscurin, cytoskeletal calmodulin and titin-interacting |  |
| p-Akt | Phosphorylated Akt |  |
| p-ANCA | Perinuclear anti-neutrophil cytoplasmic antibodies |  |
| PCA | Posterior cerebral artery |  |
| PCNA | Proliferating cell nuclear antigen |  |
| *PCNT* | Pericentrin |  |
| PCoA | Posterior communicating artery |  |
| (q)PCR | (Real-time quantitative) polymerase chain reaction |  |
| PDGFB | Platelet-derived growth factor subunit B |  |
| PDGF(-BB)/*PDGFB* | Platelet Derived Growth Factor Subunit B |  |
| PDGFRB | Platelet-derived growth factor receptor beta |  |
| *PDGFRB* | Platelet-derived growth factor receptor beta |  |
| PDI | Pressure drop indicator |  |
| PET | Positron emission tomography |  |
| PI3K/Akt/mTOR | Phosphatidylinositol 3-kinase/Akt/mammalian target of rapamycin |  |
| PKR | Protein kinase R |  |
| POA | Parieto-occipital artery |  |
| PPA | Posterior pericallosal artery |  |
| PPARγ | Peroxisome proliferator activated receptor gamma |  |
| *PPARG* | Peroxisome proliferator-activated receptor gamma |  |
| PIVH | Primary intraventricular hemorrhage |  |
| PTP1B | Tyrosine-protein phosphatase non-receptor type 1 |  |
| *PTPN11* | Protein Tyrosine Phosphatase Non-Receptor Type 11 |  |
| pVHL | Von Hippel-Lindau disease tumor suppressor |  |
| Ras-Raf-MEK-ERK | Rat sarcoma/rat fibrosarcoma/mitogen-activated protein kinase kinase/extracellular signal-regulated kinase |  |
| RING | Really interesting new gene |  |
| RNA | Ribonucleic acid |  |
| RNF213 | E3 ubiquitin-protein ligase RNF213 |  |
| *RNF213* | Ring finger protein 213 or moyamoya disease 2 |  |
| ROS | Reactive oxygen species |  |
| RSPO3 | R-spondin3 |  |
| SDF-1(α) | Stromal cell-derived factor 1 (alpha) |  |
| *SETD5* | SET domain containing 5 |  |
| sGC | Soluble guanylyl cyclase |  |
| SMC | (Vascular) smooth muscle cell |  |
| SNP | Single-nucleotide polymorphism |  |
| *SOS1* | SOS Ras/Rac guanine nucleotide exchange factor 1 |  |
| SPC | (Vascular) smooth muscle progenitor cell |  |
| STA | Superficial temporal artery |  |
| STAT | Signal transducer and activator of transcription |  |
| SULT1E1 | Sulfotransferase 1E1 |  |
| TAAD | Thoracic aortic aneurysms and dissections |  |
| TCN2 | Transcobalamin-2 |  |
| *TCN2* | Transcobalamin 2 |  |
| *TEK/TIE2* | TEK Receptor Tyrosine Kinase |  |
| TFAM | Transcription factor A, mitochondrial |  |
| TGF(-β) | Transforming growth factor (beta) |  |
| *TGFB1* | Transforming growth factor beta 1 |  |
| Th1(2) | Mature helper T cell |  |
| Tie | Tie receptor tyrosine kinases |  |
| TIMP | Tissue inhibitor of metalloproteinases |  |
| *TIMP2* | Tissue inhibitor of metalloproteinases 2 |  |
| TLR | Toll-like receptor |  |
| TNF-α | Tumor necrosis factor alpha |  |
| TNE | Transient neurological event |  |
| TSP | Thrombospondin |  |
| VCAM-1/*VCAM1* | Vascular cell adhesion molecule 1 |  |
| VEGF | Vascular endothelial growth factor |  |
| VEGFA | Vascular endothelial growth factor A |  |
| VEGF(R) | Vascular endothelial growth factor (receptor) |  |
| VSMC | Vascular smooth muscle cell |  |
| WES | Whole exome sequencing |  |
| Wnt | Wingless and Int-1 |  |
| *Xq28* (*DUPXQ28*) | Chromosome Xq28 Duplication Syndrome |  |
| *ZXDC* | ZXD Family zinc finger C |  |
|  |  |  |
